# Thiopurine Derivative-Induced Fpg/Nei DNA Glycosylase Inhibition: Structural, Dynamic and Functional Insights

**DOI:** 10.3390/ijms21062058

**Published:** 2020-03-17

**Authors:** Charlotte Rieux, Stéphane Goffinont, Franck Coste, Zahira Tber, Julien Cros, Vincent Roy, Martine Guérin, Virginie Gaudon, Stéphane Bourg, Artur Biela, Vincent Aucagne, Luigi Agrofoglio, Norbert Garnier, Bertrand Castaing

**Affiliations:** 1 Centre de Biophysique Moléculaire, UPR4301 CNRS, rue Charles Sadron, CEDEX 2, F-45071 Orléans, France; charlotte.rieux44@yahoo.fr (C.R.); stephane.goffinont@cnrs-orleans.fr (S.G.); franck.coste@cnrs-orleans.fr (F.C.); julien.cros@cnrs-orleans.fr (J.C.); martine.guerin@univ-orleans.fr (M.G.); virginie.gaudon@cnrs-orleans.fr (V.G.); biela.artur@gmail.com (A.B.); vincent.aucagne@cnrs-orleans.fr (V.A.); 2 Institut de Chimie Organique et Analytique, UMR7311 CNRS-Orleans University, Université d’Orléans, Pôle de Chimie, rue de Chartres, F-45100 Orléans, France; tber.zahira@yahoo.com (Z.T.); stephane.bourg@cnrs-orleans.fr (S.B.); luigi.agrofoglio@univ-orleans.fr (L.A.); 3 Université d’Orléans, UFR Sciences et Techniques, rue de Chartres, 45100 Orléans, France

**Keywords:** BER, DNA glycosylase, Fpg/Nei, hNeil1, disulfide, cyclophane, DNA repair inhibitors, zinc finger oxidation

## Abstract

DNA glycosylases are emerging as relevant pharmacological targets in inflammation, cancer and neurodegenerative diseases. Consequently, the search for inhibitors of these enzymes has become a very active research field. As a continuation of previous work that showed that 2-thioxanthine (2TX) is an irreversible inhibitor of zinc finger (ZnF)-containing Fpg/Nei DNA glycosylases, we designed and synthesized a mini-library of 2TX-derivatives (TXn) and evaluated their ability to inhibit Fpg/Nei enzymes. Among forty compounds, four TXn were better inhibitors than 2TX for Fpg. Unexpectedly, but very interestingly, two dithiolated derivatives more selectively and efficiently inhibit the zincless finger (ZnLF)-containing enzymes (human and mimivirus Neil1 DNA glycosylases hNeil1 and MvNei1, respectively). By combining chemistry, biochemistry, mass spectrometry, blind and flexible docking and X-ray structure analysis, we localized new TXn binding sites on Fpg/Nei enzymes. This endeavor allowed us to decipher at the atomic level the mode of action for the best TXn inhibitors on the ZnF-containing enzymes. We discovered an original inhibition mechanism for the ZnLF-containing Fpg/Nei DNA glycosylases by disulfide cyclic trimeric forms of dithiopurines. This work paves the way for the design and synthesis of a new structural class of inhibitors for selective pharmacological targeting of hNeil1 in cancer and neurodegenerative diseases.

## 1. Introduction

DNA constitutive elements are continually subjected to the deleterious effects of physical and chemical agents from endogenous and environmental sources. Induced DNA structural/chemical changes interfere with DNA transactions, such as replication, transcription and recombination [1]. Reactive oxygen species (OH·, O_2_^-^·, ^1^O_2_, H_2_O_2_, etc.), resulting from cell respiratory metabolism and inflammatory processes or from water radiolysis by ionizing radiation, Fenton reaction or photo activation processes, are responsible for the formation of numerous oxidation/degradation products of nucleobases such as the miscoding abasic site and 8-oxoguanine [2,3,4]. Oxidized bases can mislead or block replication and transcription machinery and result in mutations or cell death. These structural DNA changes initiate inflammation, carcinogenesis and age-related neurodegenerative processes [5,6,7,8]. To counteract these adverse effects, organisms, bacteriophages and viruses have evolved numerous DNA repair strategies in which the basic principles have been conserved during evolution [9]. Among these strategies, the major way to repair oxidized bases is the base excision repair (BER) pathway [10]. BER is initiated by DNA glycosylases that recognize and remove base lesions. The resulting abasic (AP) site can be excised by the combined action of AP endonucleases, AP lyases and dRp lyases. These reactions lead to one or more nucleotide gap. Finally, a DNA polymerase and a DNA ligase cooperate to fill in the gap and restore the DNA integrity.

In addition to being involved in the maintenance of genetic material (DNA repair), DNA glycosylases are also key enzymes involved in a myriad of other physiological processes [11]. They participate in the maturation of immunoglobulin antigenicity (somatic hyper-mutagenesis [SM] and class-switch recombination [CSR] via uracil-DNA glycosylase), the maintenance of telomeres (via the DNA glycosylases Neil3) and in enzymatic mechanisms of active DNA demethylation (via the DNA glycosylases SMUG1, TDG and MBD4). Human DNA glycosylases, such as hOgg1 and hNeil1, are emerging as new pharmacological targets for small-molecule modulators, given their role in a wide range of physiological and/or possible pathological processes. In Huntington disease (HD), Ogg1 (and/or Neil1)-initiated repair of 8-oxoguanine (8-oxoG, or oxidized pyrimidines) in CAG triplets is proposed to trigger iterative oxidation–excision cycles that contribute to the somatic instability of the *huntingtin* gene, through a CAG repeat expansion [12,13,14,15]. Strikingly, somatic CAG repeat instability in HD is highest in the striatum, the tissue preferentially affected by the disease, and unbalanced BER enzyme activities seems to be responsible for the tissue-selectivity of the disease [13]. Thus, selective Ogg1/Neil1 inhibitors directed in the striatum might prevent CAG repeat expansion. In another example, a small interfering RNA (siRNA)-screening approach highlighted synthetic lethal interactions between the thymidylate synthase (TS) pathway and several human DNA glycosylases (hOgg1, hNeil1) in osteosarcoma cells [16]. In a more recent study, a new mechanism has been proposed to sustain proliferation in RAS transformed cells through increased BER capability [17]. In such a mechanism, RAS-transformed cells use hOgg1 stimulation to overcome the anti-proliferative effects of excessive oxidative DNA damage. All these observations may provide new therapeutic windows in cancer therapy that might be exploited with selective drugs that specifically target Ogg1 and Neil1.

While recent studies have demonstrated the relevance of the research to design innovative anticancer strategies, only a few reported the search for hOgg1 and hNeil1 inhibitors [18,19,20,21]. In previous work, we initiated this study on DNA glycosylases from the structural Fpg/Nei superfamily [18,22,23]. These enzymes recognize and excise oxidized bases in DNA by catalyzing the cleavage of the *N*-glycosydic bond between the damaged base and its associated sugar. These enzymes are bifunctional DNA glycosylase associated with an AP lyase activity that involves the successive cleavages of phosphodiester bonds at 3′ and 5′ sides of the resulting AP site by a βδ-elimination mechanism. Fpg/Nei DNA glycosylases are composed of two globular domains, an N-terminal domain rich in beta-structures and a C-terminal domain rich in alpha-structures. All enzymes display a helix-two turns-helix (H2TH) motif that is involved in DNA binding. Most of them contain an original zinc finger (ZnF) motif (defining a new class of ZnF) comprising a β-hairpin structure with generally four cysteine residues coordinating the Zn^2+^ ion (type –C-X_2(3)_-C_(H)_-X_16-18_-C-X_2_-C-) [24,25,26]. A small number of enzymes, such as hNeil1 and mvNei1, lack ZnF and, instead, have a very similar β-hairpin motif free of zinc (called zincless finger, [ZnLF]) [27,28]. Site directed mutagenesis and crystal structures of enzymes bound to damaged DNA show that ZnF and ZnLF play exactly the same role in DNA binding. Although structurally related and displaying a slight overlap in substrate specificity, the enzymes from the Fpg subfamily remove oxidized purines, whereas those from the Nei subfamily are more specific for oxidized pyrimidines. Only the bacterial Fpg enzymes can excise 8-oxoG, the major oxidation product of purines. Thus, they are considered to be the functional homologs of the structurally unrelated eukaryote 8-oxoguanine-DNA glycosylase 1 (Ogg1) [29].

In an effort to exploit the extrahelical base lesion recognition mechanism used by these enzymes for removing oxidized bases, 2-thioxanthine (2TX) was identified among other purine and pyrimidine derivatives as an inhibitor of the bacterial Fpg and Nei enzymes and human hNeil2 [18,30]. Surprisingly, enzyme kinetic experiments with the *Escherichia coli* Fpg protein proposed an uncompetitive inhibition mode. In other words, the effective inhibitor target is probably not the active site of the enzyme. According to the uncompetitive inhibition mode, 2TX only binds the enzyme/substrate complex. This interaction is favored by prior binding of the enzyme to its DNA substrate. In fact, we demonstrated that both free and bound enzymes are targets for 2TX, with a slight preference for the bound enzyme (compatible with mixed inhibition rather than an uncompetitive or non-competitive inhibition). Studies in solution coupled with crystal structure analysis revealed that two ZnF cysteine residues are possible targets for 2TX. This effect results in the loss of zinc (observed both in solution and in crystal structures), the covalent attachment of 2TX to cysteine by a disulfide bond and, thus, the irreversible inhibition of the enzyme. Other 2TX enzyme target sites, however, are not excluded, but the irreversible character of the inhibition at a high 2TX concentration compromises the correct interpretation of enzymatic kinetics data. Although the ZnF oxidation mechanism mediated by 2TX remains unclear, it does explain why hNei1, which lacks a ZnF, is resistant to 2TX and why a strong disulfide reducer, such as tris(2-carboxyethyl)phosphine hydrochloride (TCEP), protects the ZnF-containing enzymes from the 2TX inhibitory effect [18].

In this work, we synthetized a small library of 2TX derivatives and evaluated their effects on bacterial LlFpg (from *Lactococcus lactis*) and eukaryotic Neil1 models (the human hNeil1 and mimivirus mvNei1 DNA glycosylases). We identified inhibitors that are more efficient than 2TX for the ZnF-containing Fpg/Nei DNA glycosylases. Surprisingly, some of them inhibited ZnLF-containing Nei-like proteins, mainly hNeil1 and mvNei1. Based on chemical, biochemical, mass spectrometry, computational and structural approaches, we decipher at the atomic level the mechanism of action of best inhibitors of the ZnF-containing enzymes (LlFpg) and propose a possible inhibitory mechanism for the enzymes of the Fpg/Nei superfamily that do not contain ZnF.

## 2. Results and Discussion

### 2.1. Inhibition of the Bacterial Fpg Activity by 2TX Derivatives

The compound 2TX was identified in a DNA base analog screening as an uncompetitive inhibitor of the *E. coli* formamidopyrimidine-DNA glycosylase (EcFpg) [30]. We confirmed the inhibitory effect of 2TX on ZnF-containing enzymes from the Fpg/Nei DNA glycosylase structural superfamily (including LlFpg, EcNei and hNeil2) [18]. Although the precise mode of action of 2TX remains to be clarified, we established in solution and by X-ray analysis that—unexpectedly—2TX chemically and selectively targets the two most exposed cysteine residues of the ZnF in these enzymes. Consequently, 2TX covalently attaches to cysteine through a disulfide bond, and the zinc ion is released [18]. In order to find more selective and efficient inhibitors, and to clarify the inactivation mode through the thiol/thione group, we prepared a mini-library of 2TX-derivatives (TXn) (see Supplementary Information for their synthesis and Figure S1 for their structures).

**Figure 1:**
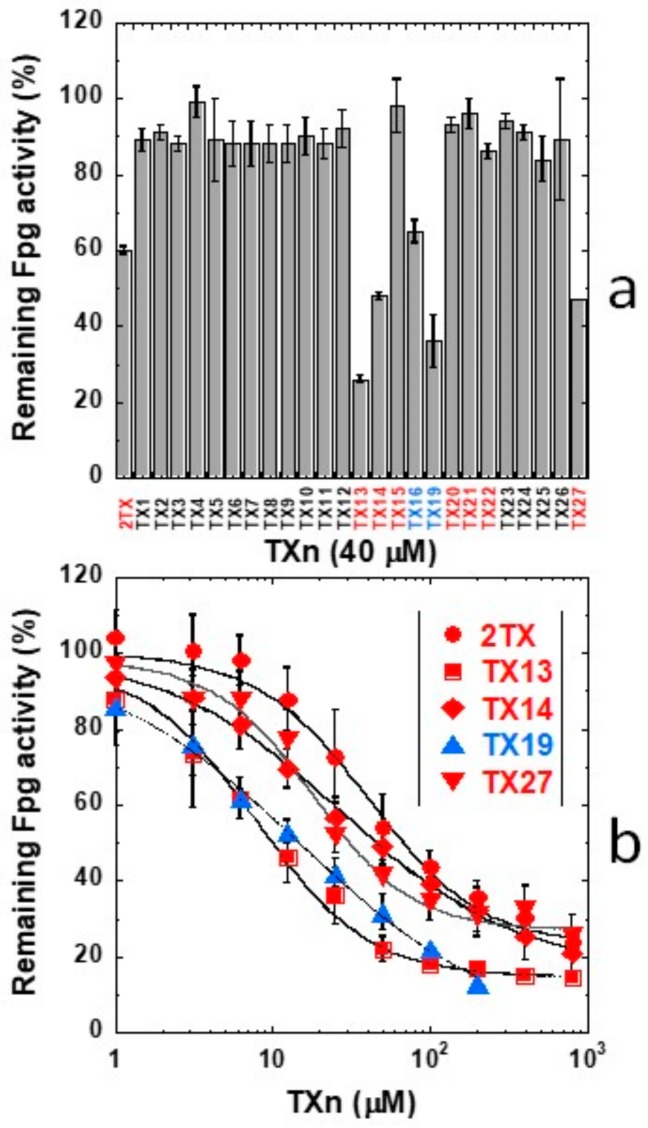
Effect of 2-thioxanthine (2TX)-derivatives on Fpg activity. A total of 20 nM of 5′-[^32^P]-8-oxoG-containing 24-bp DNA duplex (labeled on the damaged strand) was incubated with 2 nM of LlFpg alone (dimethyl sulfoxide (DMSO) control) or with the indicated 2TX and 2TX-derivatives (TXn) concentrations, as described in the “Materials and Methods” section. (**a**) Screening of TXn for Fpg inhibition. Abbreviations and structures of TXn are presented in Figure S1. Averages and standard deviations were obtained from at least three independent experiments. Mono- and di-thiolated compounds are highlighted in red and blue, respectively. (**b**) Dose-response curves for best inhibitors. Each data point represents the mean value obtained from at least three independent measurements. Half-maximal inhibitory inhibition (IC50) values (IC50_app_^A^) of the more efficient TXn are indicated in Table 1 and were extracted from these data using Origin software.

TXn were screened for their ability to inactivate the 8-oxoG-DNA glycosylase/AP lyase activity of LlFpg (our Fpg model for X-ray structure investigations). Some of these compounds are thiol-free and the others are monothiolated or dithiolated compounds (Figure S1). As expected, the compounds without the thiol/thione group were unable to efficiently inhibit the excision of 8-oxoG-containing DNA by LlFpg (Figure 1a). However, the presence of a thiol/thione group on the tested compound seemed insufficient to inhibit the enzyme. Indeed, 40 µM of some thio-compounds, such as TX15, TX20, TX21 and TX22, had a very low or unmeasurable effect on the Fpg 8-oxoG-DNA glycosylase/AP lyase activity (Figure 1a). The apparent half-maximal inhibitory concentration (IC50_app_^A^) for the best compounds were obtained from dose-response curves (Figure 1b and Table 1).

The two tested dithio-compounds TX16 and TX19 inhibited Fpg in the same range of magnitude as efficient monothio-compounds. This finding indicates a non-cumulative effect of thiol functions for Fpg inhibition (Table 1). The best inhibitor, TX13 (2-trifluoromethyl-6-thiopurine), is a monothio-compound; it was seven times more effective than 2TX, with an IC50_app_^A^ of 7.5 µM (Table 1). The 2- trifluoromethyl group of TX13 might be responsible for a better interaction of the compound with Fpg because its replacement by a methyl group in TX4 drastically decreased the inhibitory effect despite the presence of the thiol/thione group (Figure 1a). The position of the thio-group (thione/thiol) does not appear essential for inhibition and can occupy positions 2, 6, 8, 2 and 6, or, 2 and 8 on the purine aromatic ring of TXn effective inhibitors. As already shown for 2TX [18], TCEP (a strong reducer of disulfide bonds) counteracted the inhibitory effect of TXn (Figure S2).

The purine moiety of efficient TXn compounds seems to be crucial because the thio-compound TX21 did not inhibit Fpg (Figure 1a and Figure S1). Considering that Fpg repairs oxidized purines, this last observation suggests that TXn might interact with the enzyme active site. Kinetic experiments with LlFpg in the presence of 2TX partially support this last hypothesis (Figure S3). Although LlFpg was inhibited by 2TX, as was its *E. coli* homolog (EcFpg), the raw kinetic data from all the experiments appeared to be very complicated and difficult to interpret. This outcome suggests that several inhibitory mechanisms concomitantly contribute to the final inhibitory process. In previous work, we identified an irreversible inhibitory mechanism that resulted in the oxidation of the ZnF in the protein. In this case, no inhibitory constant (Ki) can be determined. This mechanism (also supported by the inhibition suppression by TCEP, Figure S2) is likely superimposed on other inhibitory mechanisms, a factor that makes it difficult to analyze kinetic data. To clarify, we separately analyzed kinetic data obtained for low and high 2TX concentrations (Figure S3). As expected, we observed a linear dependence of the reciprocal velocity (1/v) as a function of the reciprocal substrate concentrations (1/S) with and without the inhibitor. The kinetic constant values for the excision of 8-oxoG by LlFpg were K_m_ = 5.60 nM, V_max_ = 0.34 fmol.min^-1^ and k_cat_ = 0.09 min^-1^. These values compare well with those determined previously for the excision of FapyG and 8-oxoG by EcFpg [31,32,33]. At low 2TX concentrations, the inhibitory process appears partially competitive, with an apparent K_i_ of 2.19 ± 0.56 µM (K_ic_). This value is consistent with an interaction of 2TX in the active site of the enzyme. This inhibitory mechanism has not been previously described for the EcFpg enzyme [30]. At high 2TX concentrations, the inhibition displays a clear uncompetitive mechanism (decrease of V_M_ and K_M_, their ratio remaining relatively invariant) with an apparent K_i_ of 24.88 ± 6.56 µM (K_iu_) as already observed with the EcFpg [30]. This mode of inhibition is in agreement with the possibility that 2TX can bind outside the active site on the free and bound enzyme. We will later discuss that the difficulty in interpreting the enzymatic kinetics data in the presence of 2TX (and TXn) can also be explained by the different redox equilibrium states of these compounds in dimethyl sulfoxide (DMSO), which cannot be easily controlled and adds additional complexity. The loss of the inhibition in the presence of TCEP also suggests that the reduced forms of 2TX and TXn are weakly or not effective in inhibiting the enzyme (see below).

### 2.2. Inhibition of the Bacterial Fpg DNA Binding Activity by 2TX and TXn

**Figure 2:**
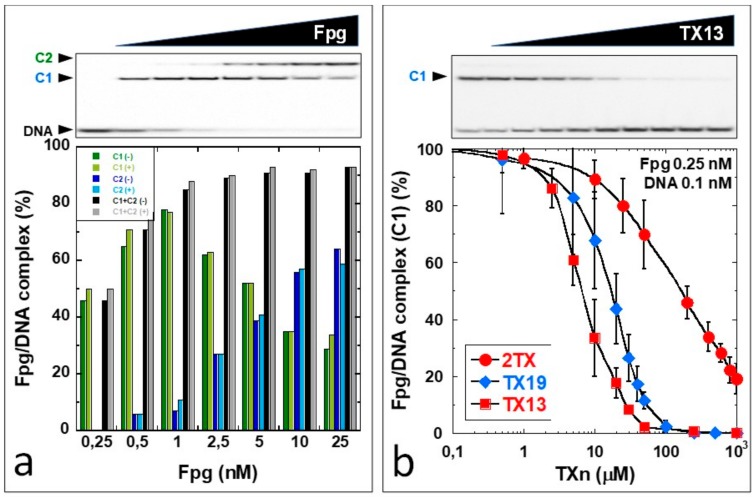
The effect of 2TX and TXn on Fpg DNA binding to abasic site-containing DNA. A 14-mer DNA duplex containing a tetrahydrofurane (THF) site as an abasic site analog was incubated with a limiting Fpg concentration (0.25 nM) with and without increasing concentrations of TXn. Incubation mixtures were then analyzed by electrophoretic mobility shift assay (EMSA), as described in the ‘Materials and Methods’. (**a**) Titration experiments with (+) and without (-) 2 mM tris(2-carboxyethyl)phosphine hydrochloride *(TCEP)* C1 and C2 are for the [1/1] and [2/1] Fpg/DNA complexes, respectively (see detailed in the text). (**b**) Fpg DNA binding concentration-dependence for 2TX and the more efficient TXn inhibitors. A representative EMSA autoradiography with 0.25 nM of Fpg alone (lane no) and 0.5, 2.5, 5, 10, 20, 40, 50, 250, 1000 µM is shown in the upper panel. IC50app^B^ corresponds to the TXn amount needed to halve the C1 complex formation and was extracted from the dose-response curves (lower panel; Table 1).

To complement this study, we examined the effect of TXn on the binding of Fpg to a short DNA duplex containing a tetrahydrofuran (THF), a non-cleavable AP site analog. The enzyme specifically recognizes THF and forms a stable abortive complex with DNA [18,34,35,36]. Qualitative and quantitative binding experiments were performed by electrophoretic mobility shift assay (EMSA), and DNA binding conditions were optimized for 8% DMSO (required for TXn dissolution but did not negatively affect enzyme stability and activity) and 0.6% bovine serum albumin (BSA; required to avoid an enzyme dilution negative effect and to obtain reproducible results; Figure S4). A representative titration experiment (with and without TCEP) of the 14-mer [THF:C] DNA duplex by LlFpg is shown in Figure 2a. Fpg formed two stable complexes with this short DNA probe that were easily observed by EMSA: a high-affinity complex C1 corresponding to the lesion recognition complex (LRC), with nanomolar affinity [34,35,37], and a complex C2 only observed at higher Fpg concentrations. C2 results in C1 titration by a second Fpg molecule. This finding illustrates that the crystal structure of Fpg bound to 8-oxoG-carbanucleotide-containing DNA [38] (Figure S5). The stability of each preformed complex was then examined by EMSA in the presence of 2TX or TXn. In these experiments, the choice to pre-form the complex before adding 2TX or TXn in the reaction mixture was based on the observation that pre-incubation of Fpg for 20 min at 4 °C with 2TX or TXn inhibitors in a concentration range varying from micromolar to millimolar completely abolished the enzyme’s ability to bind to DNA. This data unambiguously indicates that 2TX and TXn effective inhibitors can interact with the enzyme even in the absence of DNA. This result is consistent with the non-competitive inhibition of Fpg by 2TX. This mechanism was also partially suggested by enzymatic kinetic studies at a high concentration of inhibitors and by previous work (Figure S3) [18]. Indeed, this mechanism suggests that 2TX can inactivate the enzyme by indifferently interacting with the enzyme alone or with the enzyme/substrate complex outside the enzyme active site. The enzyme inactivation mediated by 2TX through ZnF oxidation that directly affects the DNA binding properties of the enzyme is also in agreement with this last observation [18]. Dose-response curves of preformed C1 in the presence of increasing thio-compound concentrations, which we showed effectively inhibit Fpg activity, induced the dissociation of the Fpg/DNA lesion recognition complex C1 (Figure 2b).

**Figure 3:**
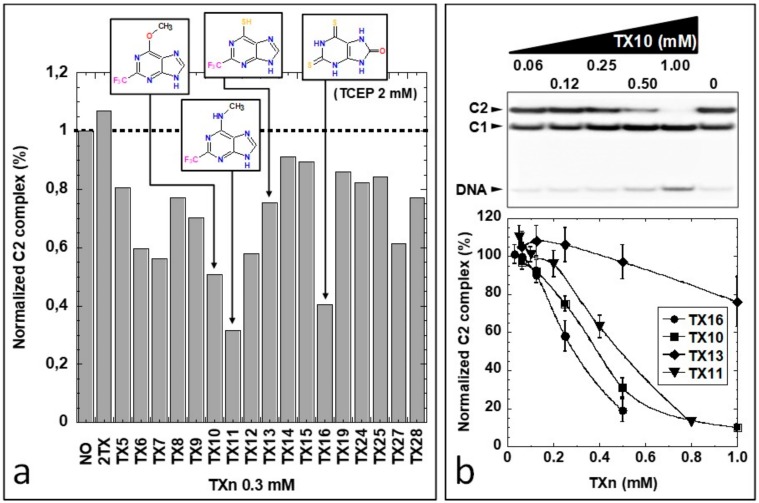
Impact of TXn on the non-specific Fpg/DNA complex C2 stability in highly reducing conditions. EMSA was performed as described in the ‘Material & Methods’, except TCEP was added at a 2 mM final concentration in each binding assay. (**a**) Representative experiment at 0.3 mM 2TX and more efficient TXn; (**b**) Dose-response with the best TXn. Upper panel: Representative EMSA experiment with TX10. Lower panel: Dose-response curves with the indicated TXn.

The inhibitor concentrations that induced the loss of the half-maximal binding (IC50_app_^B^) were determined from these curves and are reported in Table 1. IC50_app_^B^ compared well with IC50_app_^A^. As observed above for the enzyme activity, strong reducers, such as TCEP, abolished the negative effects of 2TX and TXn on Fpg/DNA complex stability and formation. These findings again indicate that inhibition by thio-compounds essentially relies on the oxidation of the Fpg ZnF (for an example, see experiment with TX19, Figure S4). Surprisingly—and for high concentrations of TXn (above 0.1 mM)—the effect of some TXn on the stability of the complex C2 (preformed complex before treatment) in the presence of TCEP was highly variable. For a [1/1] molar ratio between TCEP and TX13 or TX27, C2 was strongly destabilized, but C1 remained stable (Figure S6). Under the same conditions, 2TX, TX14, TX15 and TX19 had a moderate effect on C2. This troubling and unexpected observation led us to evaluate this effect with the TXn small library compounds. To prevent a side effect due to a possible partial reduction by TCEP, we performed EMSA in the presence of a 6.7-fold molar excess of TCEP with respect to 2TX and TXn after complex formation (Figure 3).

As expected in the presence of TCEP, thiol/thione function in TXn is not an absolute criterion because among the best C2 destabilizers, we found compounds that lack thiol group, including TX10 and TX11. On the contrary, the presence of an alkyl group (trifluoromethyl and methyl groups) at position two of the purine derivatives (TX6, TX7, TX10, TX11 and TX12, Figure S1) was favorable for destabilizing C2. These data suggest that some TXn compounds interact with Fpg (here LlFpg) with an unknown enzyme binding site. This finding results in incompetency of a second Fpg molecule to form the C2 complex with the 14-mer [THF:C] DNA duplex. Obviously, the binding of these compounds to this exosite had a limited effect on the stability of the Fpg molecule bound to the lesion (LRC, C1; Figure S5a). The crystal structure of LlFpg bound to 14-mer [8-oxoG:C] DNA duplex (which differs from that used in this study only by the damaged nature present in the duplex: an 8-oxoG lesion in place of THF) revealed that the second Fpg molecule can bind to the overhanging base positioned at the 5′ end of the damaged strand (Figure S5b) [38]. In the structure, the first Fpg molecule (Mol-1 in Figure S5a) already positioned on the base lesion, does not physically interact with the second one (Mol-2 in Figure S5a). Combining these biochemical and structural observations, we propose that some TXn can selectively bind Fpg outside the active site, at an exosite. This action impairs its ability to bind to the overhanging base at the DNA duplex end. According to this hypothesis, some TXn can mimic the overhanging base of the DNA duplex and displace it, a phenomenon that destabilizes C2. When a high affinity TXn occupies the exosite, Fpg appears unable to form the C2 complex. Molecular docking experiments and crystal structures presented below will confirm this hypothesis.

### 2.3. Selective Inhibition of ZnLF-Containing Fpg/Nei DNA Glycosylases by Dithio-TXn

**Figure 4:**
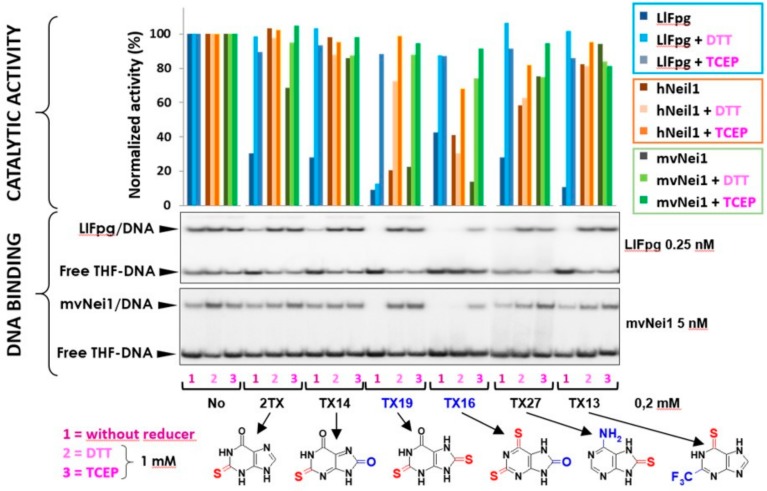
Comparative analysis of the effect of 2TX and TXn on Fpg/Nei DNA glycosylases. Enzyme activities were measured as described in the Figure 1 legend using 0.1 nM of a 5′-[^32^P]-24-mer DNA duplex containing either an 8-oxoG opposite C for LlFpg or a dihydroxyuracil (DHU) opposite G for hNeil1 and mvNei1. The DNA probe was incubated with the indicated protein concentration and 0.2 mM TXn compounds in the presence or absence of 1 mM dithiothreitol (DTT) and TCEP (a representative experiment is illustrated by the upper histograms). The DNA probe for EMSA was 0.1 nM of 5′-[^32^P]-24-mer DNA duplex containing THF with LlFpg and mvNei1 (bottom panels: gel autoradiography) with the indicated conditions and as described in ‘Materials and Methods’.

In previous work, we showed that 2TX inhibits ZnF-containing Fpg/Nei DNA glycosylases (EcFpg, EcNei, LlFpg and hNeil2) [18,30]. At high concentrations, this inhibition essentially results in ZnF oxidation associated with zinc release because the zincless (ZnLF)-containing enzyme appears resistant to 2TX. To complement this present study, we evaluated the TXn effect on the catalytic and DNA binding activities of two ZnLF enzymes: hNeil1 and mvNei1. Regarding the capacity to oxidize cysteine by the thiolated TXn, the two proteins are good complementary models: hNeil1 displays eight free cysteine residues whereas mvNei1 lacks cysteine (note that LlFpg only contains four cysteine residues, all involved in Zn^2+^ ion coordination in the ZnF motif). As anticipated, 2TX and all tested monothiolated TXn compounds did not significantly affect hNeil1 and mvNei1, as opposed to the effect on LlFpg (Figure 4).

Interestingly and unexpectedly, the two dithiolated TXn (TX16 and TX19) evaluated in the study displayed apparent inhibitory effects on both the catalytic and DNA binding activities of hNeil1 and mvNei1. One of the two thiol/thione groups substituted position two of the purine ring and the second substituted position six or eight of the purine analogs (TX16 and TX19, respectively). Although the monothiolated TXn were ineffective on these enzymes, hNeil1 inhibition might be attributed to the potential chemical reactivity of free cysteine residues but not for mvNei1, which is completely devoid of cysteine. The best inhibitor was TX16, with an IC50app^A^ of 21 and 14 µM for hNeil1 and mvNei1, respectively (Table 1). 

Based on our knowledge, TX16 is among the best inhibitors of ZnLF-containing Fpg/Nei enzymes discovered to date. In previous work, using a high-throughput screen of a small molecule library, the Lloyd group identified purine analogs with an IC50 of 10–30 µM for hNeil1 (determined by a radioassay similar to our assay) [20]. Although it is difficult to conclude the TX16 and TX19 mechanism of inhibition of hNeil1 inhibition, mvNei1 inhibition cannot be attributed to an attack of a cysteine. The simplest hypothesis to explain this result is that TX16 (TX19) interacts with mvNei1 (hNeil1) either at the active site and/or another site(s) to impair the ability of the enzyme to bind and/or metabolize its DNA substrate. This mode of inhibition might also exist with LlFpg, a hypothesis that would explain why it was difficult to extract from enzymatic kinetics a precise mode of inhibition because several mechanisms of inhibition contribute to the final inhibition observed (Figure S3).

The most astonishing finding in this study lies in the observation that strong reducers, such as TCEP and to a lesser extent dithiothreitol (DTT), suppressed the inhibition of the hNeil1 and mvNei1 DNA binding and catalytic activities mediated by TX16 and TX19 (Figure 4). If we consider that TCEP is the more powerful reducing agent to break disulfide bond (as compared to DTT) and that the cysteine-free enzyme mvNei1 was inhibited by TX16 and TX19, the simplest hypothesis to explain this last observation is to propose that the reducer has a direct action on enzymes (protein oxidation protection) as well as on TX16 and TX19. According to this proposition, we suggest that disulfide forms (and not other possible oxidized forms) of TX16 and TX19 are potentially responsible for the inhibition of hNeil1 and mvNei1. Due to the presence of two thiol/thione groups in TX16 and TX19, we cannot exclude the possibility of having many oligomeric forms of these compounds in our stock solutions. 

### 2.4. Molecular Mechanisms for Fpg/Nei Inhibition by Disulfide forms of 2TX and TXn

**Figure 5:**
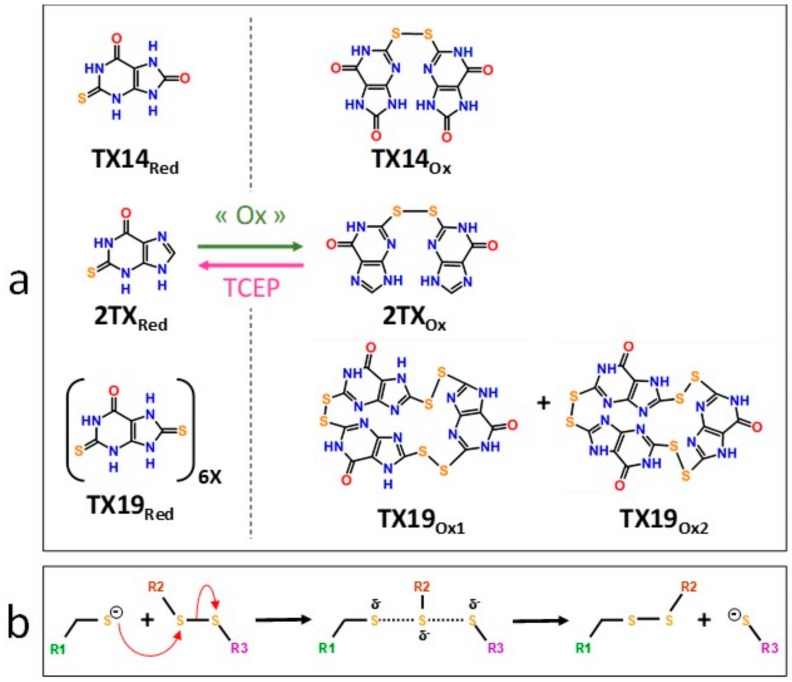
Disulfide forms of 2TX, TX14 and TX19 are responsible for the inhibition of Fpg/Nei DNA glycosylases. (**a**) Disulfide forms of 2TX, TX14 and TX19 identified by LC-HRMS (see the mass spectra in Figure S7). (**b**) Proposed thiol/disulfide exchange mechanism for the inhibition of zinc finger-containing Fpg/Nei enzymes by disulfide forms of 2TX, TX14 or TX19. R1 indicates cysteine thiolate-containing zinc finger Fpg/Nei enzyme, R2 and R3 belong to disulfide form of thio-compounds and can be identical or different.

The simplest way to evaluate the molecular homogeneity of the 2TX and TXn stock DMSO solutions used in this study is content analysis by mass spectrometry (MS). Given that direct analysis of samples by matrix-assisted laser desorption/ionization-time of flight (MALDI/TOF) or electrospray ionization (ESI) MS is impossible due to the presence of DMSO, we used high-resolution ESI-MS coupled with liquid chromatography separation (LC-HRMS). LC-HRMS analysis was performed with stock solutions of the monothio 2TX and TX14 and the dithio TX19 (Figure S7). The 2TX and TX14 stock solutions unambiguously contained two major compounds: one having the expected mass of their reduced forms (Figure 5a, 2TX_Red_, TX14_Red_) and a second one with a mass consistent with their corresponding disulfide dimeric forms (Figure 5a, 2TX_Ox_ and TX14_Ox_). The LC-HRMS spectrum of TX19 displays a more complex LC-profile, a finding that indicates a greater heterogeneity of this compound in DMSO. The molecular species eluted in peaks one and two may correspond to a reduced form of TX19 that lost one sulfur atom (∆mass= −17 u.m.). The compound eluted in peak three corresponds to the reduced form of TX19 (Figure 5a, TX19_Red_). Two other compounds were later eluted separately later and display the same molecular mass expected for the two cyclic trimeric disulfide isomers of TX19 (Figure 5a, TX19_Ox1_ and TX19_Ox2_).

The diversity of TX19 oxidized species present in the DMSO stock solution appears to be restricted to the two possible cyclic isomers of the trimeric disulfide species (no disulfide linear species, such as dimers, trimers and other oligomers; no other cyclic oligomeric disulfide oxidized species involving less or more than three monomers). The possibility of having multiple redox states in 2TX and TXn stock solutions raises the question of which form(s) of the molecule is(are) responsible(s) for the Fpg/Nei DNA glycosylase inhibition we observed. Previous experiments strongly suggest the involvement of disulfide forms of 2TX and TXn in the inhibition of Fpg/Nei DNA glycosylases because TCEP completely abolished the inhibition of all the enzymes tested in this work (Figure 4).

**Figure 6:**
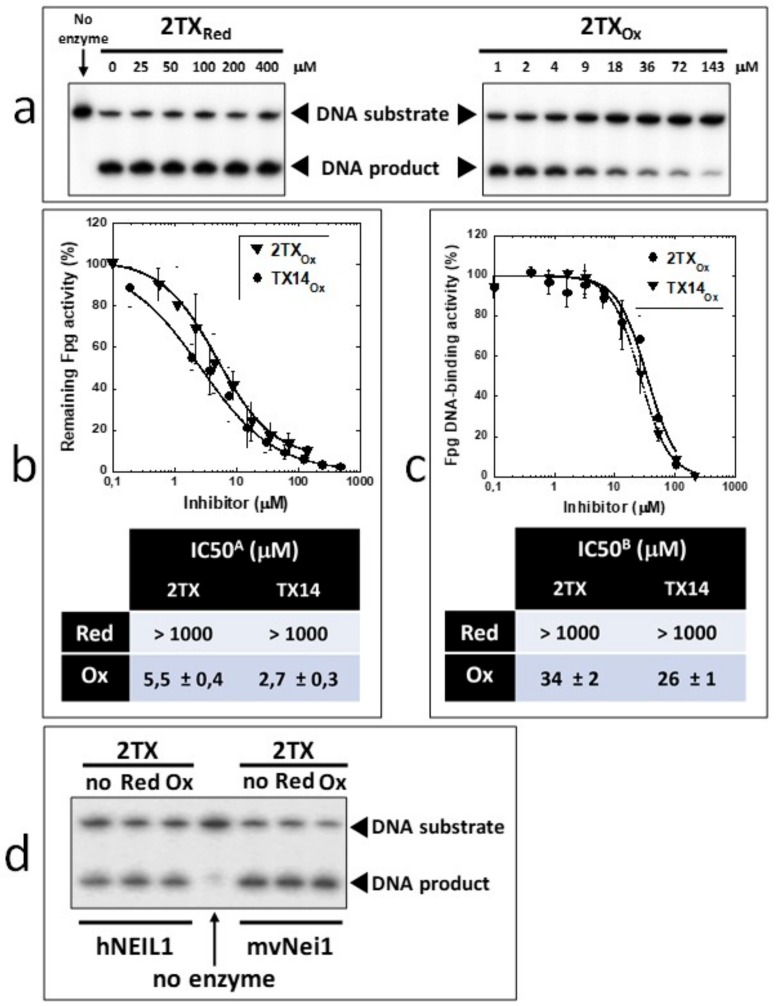
Comparative analysis of the effect of disulfide forms of 2TX and TX14 on the Fpg/Nei DNA glycosylases. Homogeneous reduced and disulfide forms of 2TX and TX14 were freshly prepared at 10 in 50 mM sodium phosphate (pH8.0). Catalytic and DNA binding activities of Fpg/Nei DNA glycosylases were performed as described in the ‘Materials and Methods’ using the radiolabeled [8-oxoG:C] and [DHU:C] DNA duplexes as substrate for the Fpg and hNeil1 (mvNei1) catalytic activity, respectively, and the 14-mer [THF:C] DNA duplex for DNA binding activity of all enzymes. IC50^A^ and IC50^B^ were determined from three independent experiments. (**a**) Representative autoradiography of 8-oxoG-DNA incubated with Fpg in the presence of the indicated 2TX_Red_ and 2TX_Ox_ concentrations. **(b)** and **(c)** dose-response curves and IC50 determination for Fpg catalysis and DNA binding, (**d**) the effect of 2TX_Red_ and 2TX_Ox_ on the hNeil1 and mvNei1 activity.

To confirm this hypothesis, we established experimental procedures to prepare the homogeneous disulfide forms of the monothio-compounds 2TX and TX14 by using sodium periodate (NaIO_4_), a more appropriate oxidant that we used to produce disulfide species rather than other oxidized species (Figures S8 and S9). To avoid DMSO-mediated oxidation, the dry powders of the homogeneous reduced and oxidized species were dissolved in 50 mM sodium phosphate, pH8.5. The effect of each homogeneous redox form of these compounds was then evaluated separately on the catalytic activity and DNA binding properties of LlFpg (Figure 6). Only the disulfide forms 2TX_Ox_ and TX14_Ox_ inhibited Fpg catalytic activity, with an IC50 of 5.5 and 2.7 µM, respectively (right panel of Figure 6a,b). By contrast, 400 µM of 2TX_Red_ and TX14_Red_ did not inhibit the enzyme (left panel, Figure 6a). The IC50^A^ values for activity were approximatively 10 times greater than the IC50_app_^A^ determined with the redox mixtures in DMSO (Table 1). We drew similar conclusions with regard to the effect of disulfide of 2TX and TX14 forms on the DNA binding properties of LlFpg (IC50^B^, Figure 6c). The IC50^B^ obtained for homogeneous species was always 6 to 10 times greater than the IC50^A^, data that suggest the catalytic activity and DNA binding experimental conditions were too different to be compared (incubation temperature, DNA probe and protein concentrations, etc.). Nevertheless, the two measured activities varied in the same direction. As observed above (Table 1), TX14_Ox_ was twice as effective as 2TX_Ox_ for inhibiting LlFpg, data that again show the selectivity of TXn. As expected, given the results from experiments that use redox mixtures in DMSO, neither 2TX_Ox_ nor TX14_Ox_ inhibited hNeil1 and mvNei1 (Figure 6d). Here again, there was a selectivity of monothio compounds for proteins with ZnF. The inhibition of LlFpg by 2TX and TX14 (and by other monothiol-compounds TX13 and TX27 and dithio-compounds TX16 and TX19) surely resulted from a thiol/disulfide exchange mechanism between thiolates of ZnF cysteine and the disulfide forms of 2TX and TXn (monothio- or dithio-compounds) (Figure 5b). Even if each cysteine thiolate of ZnF is potentially involved in the thiol/disulfide exchange mechanism, we previously showed that only the more exposed Cys248 and Cys268 of the LlFpg ZnF are susceptible to form a disulfide bond with 2TX [18]. However, we cannot completely exclude that a similar mechanism participates in the inhibition of hNeil1. Notably, we failed to observe TX16 and TX19 covalent adducts by mass spectrometry (MALDI-TOF) following a long incubation of hNeil1 with these compounds. The low reactivity of the free cysteine residues of hNeil1 might be linked to the fact that they are buried in the structure of the protein and, therefore, not accessible to the solvent and thus to TX16 and TX19 [27]. Obviously, a thiol/disulfide exchange mechanism is completely excluded with mvNei1, which has no cysteine. Despite many attempts, we failed to prepare the soluble and homogeneous forms TX19_Ox1_ and TX19_Ox2_. Although we have no doubt that the oxidized trimeric cyclic forms of TX19 are responsible for the inhibition of hNeil1 and mvNei1 proteins, we cannot provide experimental proof of such a process.

### 2.5. Structural Insights into the Mechanism of Fpg/Nei DNA Glycosylase Inhibition by 2TX and TXn

#### 2.5.1. Proposed Binding Sites from Blind and Flexible Docking of Reduced and Oxidized Forms of 2TX and TX19 on Free and DNA Bound LlFpg and hNeil1

**Figure 7:**
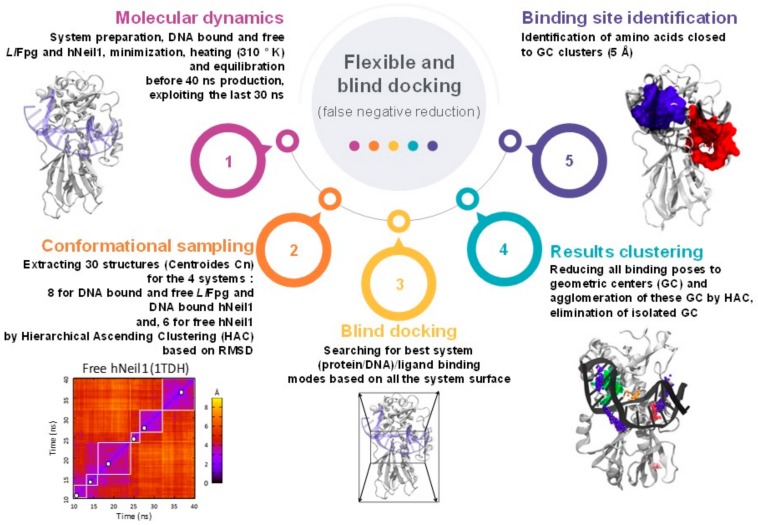
Flexible and blind docking strategy.

To exhaustively search for potential interaction sites by docking, we considered the intrinsic flexibility of receptors (free and DNA bound proteins) and ligands (conformations and tautomers) as described in Materials and Methods Section. Figure 7 presents an overview of the key steps of the blind and flexible docking procedure we used. 

**Figure 8:**
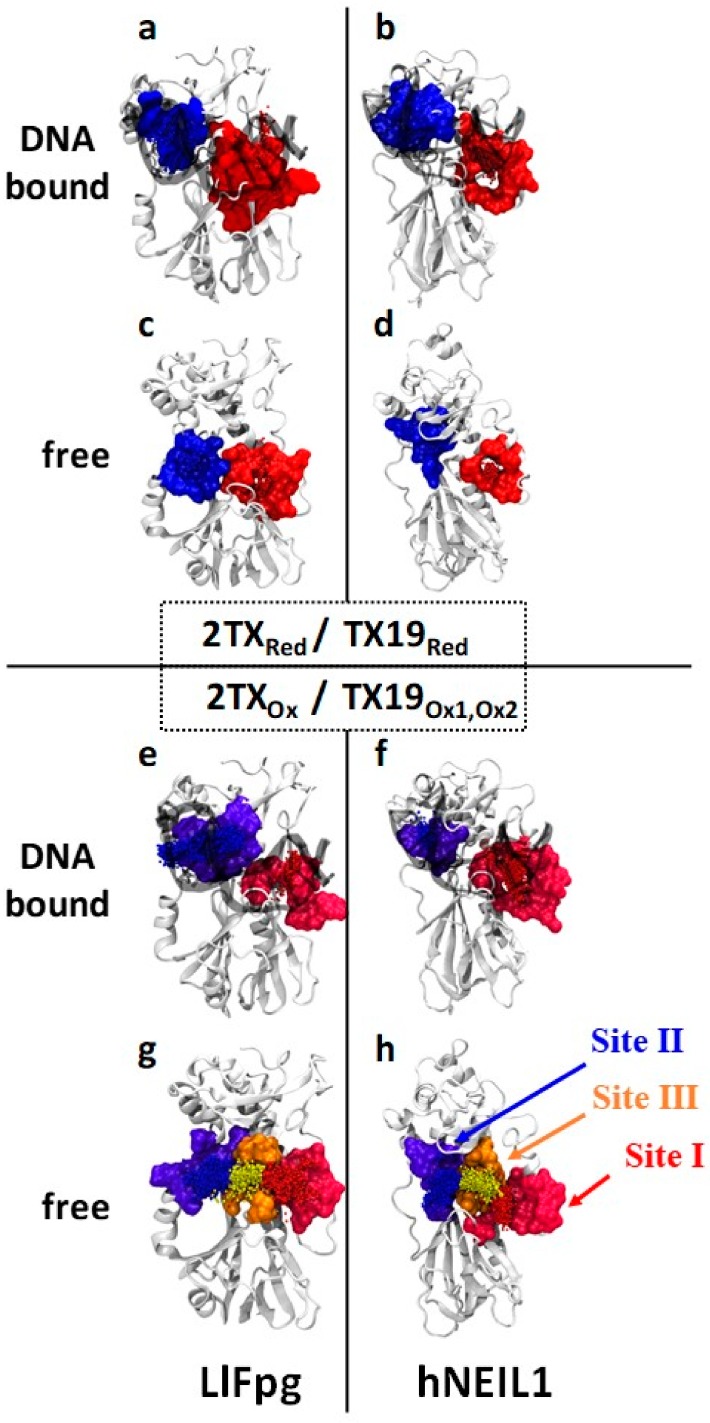
Binding sites of the 2TX and TX19 proposed by blind docking. (**a**–**d**) Docking of 2TX_red_ and TX19_red_ on free and DNA bound LlFpg and hNeil1 as indicated. (**e**–**h**) Docking of 2TX_ox_ and TX19_ox_. Sites in red, blue and orange correspond to site I, II and III, respectively. Proteins are represented by a light grey ribbons and DNA are drawn by translucent black ribbons. Small spheres indicate the geometric centers of blind docking poses of reduced and disulfide forms of 2TX and TX19.

We performed docking experiments with 2TX_Red/Ox_ and TX19_Red/Ox1/Ox2_ as ligands on free and DNA bound LlFpg and hNeil1 (for example, the docking of TX19_Ox_ conformers is described in pictures, Figure S10). With 2TX_Red_ and TX19_Red_, two Fpg/hNei1 binding sites were proposed: site I (including the active site) and site II, which mainly includes the two DNA binding motifs found in all Fpg/Nei DNA glycosylases (the ZnF or ZnLF loop that contains the strictly conserved R260 or R276 residue, respectively, and the H2TH motif, [29]) (Figure 8 and Figure S11). Binding site I is partially composed of amino acids that are directly involved in catalysis (such as the strictly conserved P1 and E2 residues) and partly in the lesion capping loop (LCL, the αF-β9 loop in which residues differ between Fpg and Nei subfamily; Figure S11, Table S1) [22,29,39]. The potential binding of the thio-compounds in the active site is consistent with the competitive inhibition mode suggested by Fpg kinetics experiments analyzed at low 2TX concentrations (see text above and Figure S3). The binding site II area is partly constituted by the strictly conserved LlFpg residues L162 and E163 localized in the helix αD of the H2TH motif and Q165 localized in the H2TH first turn (L165/M162, D167/D163 and Q168/Q164 of hNeil1/mvNei1, respectively; Figure S11). The strictly conserved arginine of the ZnF and ZnLF loop (R260 and R276 for LlFpg and hNeil1, respectively), which neutralizes the DNA phosphate groups located on both sides of the damage nucleoside, is often mobilized to interact with reduced forms of 2TX/TXn. When DNA is present in the structure, it seems that hydrophobic interactions and hydrogen bonds DNA also contribute to the binding of reduced and oxidized 2TX/TXn in site II (Figure S12). This phenomenon appears especially true for nucleotide phosphates localized at the 3′ side of the THF damaged nucleoside (see below). The zinc coordination sphere of the LlFpg ZnF was not identified as a 2TX_red_/TX19_red_ binding site by docking experiments. The binding of 2TX_red_ and TX19_red_ to site II (i.e., outside the active site) on free and DNA bound enzyme might be associated with uncompetitive or non-competitive inhibition, as suggested for LlFpg by kinetics experiments analyzed at high 2TX concentrations (Figure S3). However, the biochemical data about the effect of 2TX/TX19 in the presence of TCEP (i.e., reduced species) is not consistent with this hypothesis. This discrepancy suggests that binding of 2TX_red_/TX19_red_ to sites I and/or II would not be associated with Fpg/Nei enzyme inhibition.

**Figure 9:**
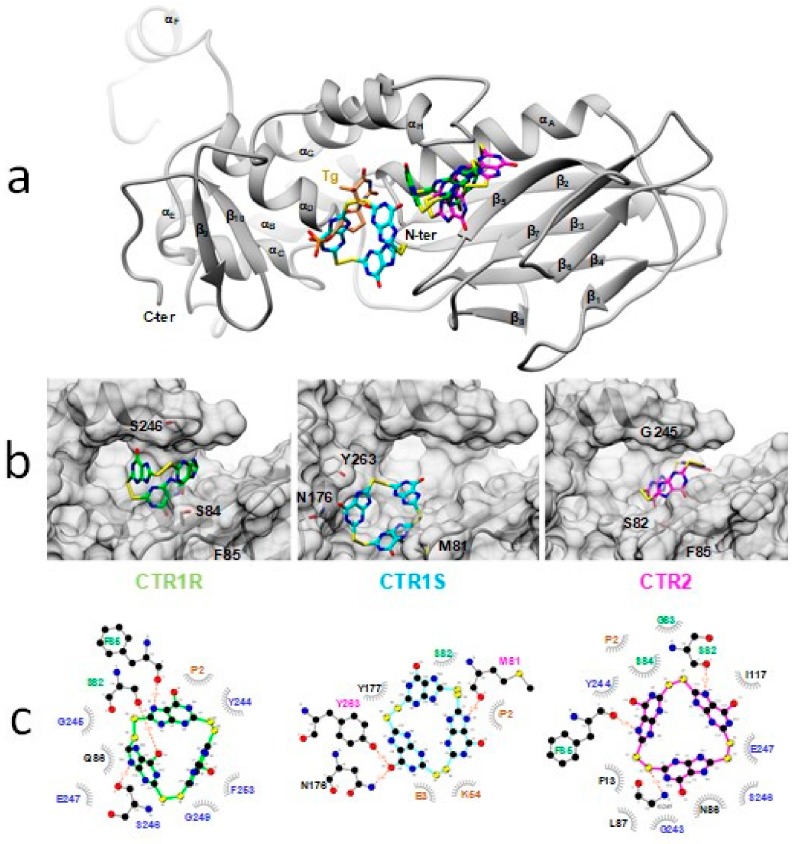
Best docking poses of the three circular trimeric disulfide forms of TX19_Ox_, CTR1R, CTR1S (TX19_Ox1_) and CTR2 (TX19_Ox2_) observed at site III of the free hNeil1 protein (**a**) Superimposition of each docked ligand on the crystal structure of hNeil1 bound to thymine glycol (Tg)-containing DNA (1ITY). For clarity, DNA contained in the crystal model has been deliberately omitted and only the protein in grey ribbon and Tg in brown are shown. This view shows the steric clash between Tg and docked ligands, especially CTR1S. (**b**) Zoom of best docked poses for the three ligands. The solvent accessible surface area in the vicinity of the docking site of hNEIL1 is shown in grey. (**c**) 2D LIGPLOT representations that show protein residues mobilized to stabilize each docked ligand in site III. Hydrogen bonds between protein residues and ligands are shown by red dashed lines and hydrophobic interactions by grey arcs of circle. Catalytic residues of hNeil1 are highlighted in orange. Residues of hNeil1 known to interact directly with the flipped oxidized pyrimidine Tg are colored in magenta. Residues in blue are involved in the lesion capping loop that is essential for stabilizing the flipped damaged nucleoside inside the active site pocket, and residues in green are involved in the intercalation loops of the protein inserted in the DNA minor groove in the hole resulting from the flip out of the damaged nucleoside (Figure S11b). A similar observation can be made for the free LlFpg.

To complement the biochemical data that showed only the disulfide forms of 2TX and TX19 are responsible for enzyme inhibition (Figure 6), we performed blind docking experiments with 2TX_ox_ and TX19_ox_ disulfide forms (Figure 6a, Figures S10 and S12). They revealed two major binding sites on both free and DNA bound enzymes that correspond to binding sites I and II already identified with 2TX_red_ and TX19_red_ (Figure 8e,f). Interestingly, a third TX19_ox_ binding site (named binding site III), which is located between sites I and II, is also proposed by docking experiments, but only on the free enzymes (Figure 8g,h, Figure 9 and Figure S10). Amino acid residues that line the binding site III are listed in Table S2, and some of them belong to binding sites I or II. Binding site III is hidden when the Fpg/Nei enzymes are bound to DNA (Figure 8e,f). Based on these data, we propose that LlFpg and hNeil1 inhibition by TX19 can result from the binding of its disulfide forms (cyclic trimeric forms, Figure 6a) to site III. The occupation of site III by TX19_ox_ might prevent DNA binding and thus catalysis. The best docking poses in site III of TX19_Ox_ (CTR1R, CTR1S and CTR2) on free enzymes clash with DNA present in the crystal structure of hNeil1 bound to thymine glycol (Tg)-containing DNA (Figure 9). In particular, CTR1S superimposes with flip out Tg base damage in the hNeil1 LRC complex [28]. Thus, docking experiments can provide a reason for the inhibition of hNeil1 and mvNei1 by the dithio-compounds TX16 and TX19 (Figure 4 and Table 1).

#### 2.5.2. Crystal Structures of Thiopurine Derivatives Bound to Fpg/DNA Complex Partially Confirm Docking Experiments

**Figure 10:**
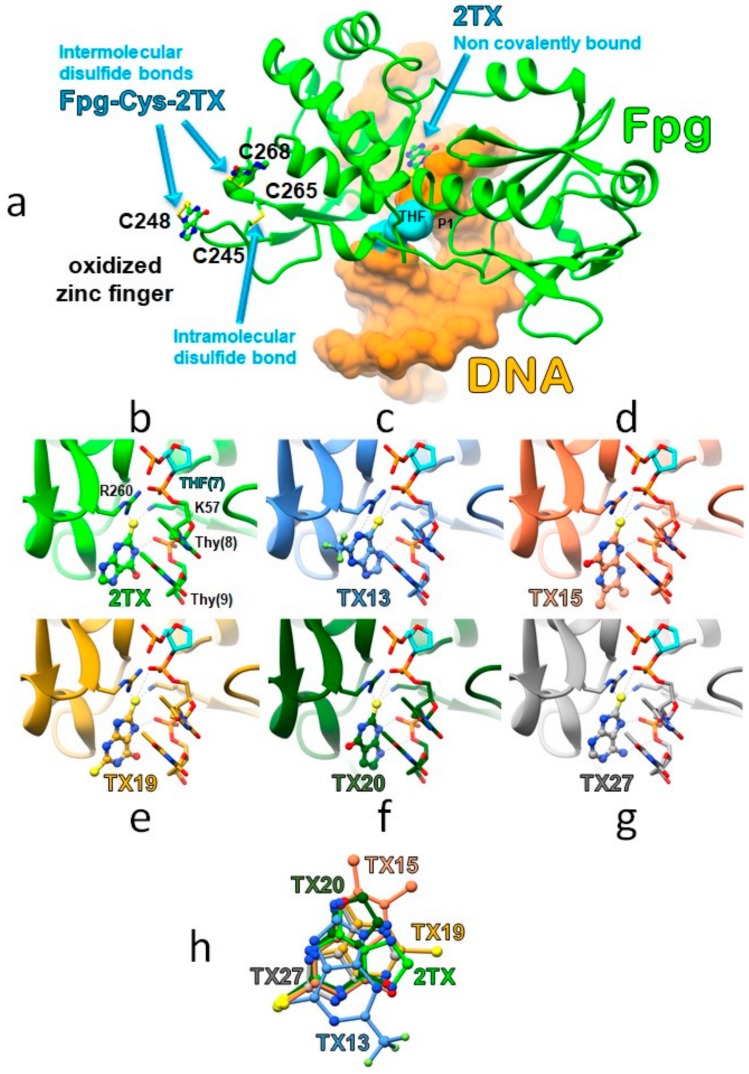
Crystal structures of LlFpg bound to 14-mer [THF:C] DNA in the presence of 2TX/TXn. (**a**) Overview of the structure with 2TX, (**b**) to (**g**) zoom of the non-covalent binding site (i.e., binding site II) with 2TX, TX13, TX15, TX19, TX20 and TX27, respectively, in the absence of TCEP and, (**h**) superimposition of 2TX/TXn inside the LlFpg non-covalent binding site based on a Cα peptide backbone alignment of the protein. The THF abasic site analog is highlighted in magenta. The N-terminal active site proline 1 (P1) is indicated. Ball-and-stick model was used for representing 2TX and TXn. Sulfur atoms are indicated by yellow balls and the intra- and inter-molecular disulfide bonds by yellow sticks. The direct interactions between thiopurines and the side chain of Fpg residues K57 and R260 and between thiopurines and the DNA backbone phosphates at the 3′ side of THF are indicated by grey dashed lines.

To further our understanding of the TXn mode of action, we solved several crystal structures of LlFpg bound to 14-mer [THF:C] (LRC) in the presence of thio-compounds. To this end, crystals of the [LlFpg/DNA] complex were soaked in a saturated 2TX/TXn solution of mother liquor, as previously described [18]. Using this method, we successfully resolved the structures of LRC with the moderately potent inhibitor 2TX (new crystal structure), high-efficiency inhibitors TX13, TX19, TX20, TX27, low-efficiency inhibitor TX15 and, TX19 in the presence of TCEP (Table S1). Despite several attempts, we failed to obtain 3D structures with the efficient inhibitors TX14 and TX16, both of which harbor a C8-oxo substitution in the purine ring. In all cases, these crystal structures displayed one molecule of Fpg by the DNA duplex. We did not observe a second molecule binding to the DNA duplex extremity, as we observed with LlFpg bound to 14-mer duplex containing 8-oxoG in place of THF (Figure S5) [38]. In the absence of reducer and as previously observed with 2TX, all crystal structures showed that 2TX/TXn induced oxidation of the ZnF cysteine residues and the concomitant loss of the Zn^2+^ ion [18]. In the present structure of LlFpg/DNA with 2TX, all ZnF cysteine residues are oxidized. The two more exposed LlFpg cysteine residues (Cys248 and Cys268) form an intermolecular disulfide bridge with one 2TX molecule and the two other cysteine residues (Cys245 and Cys265) establish an intramolecular disulfide bond between them (Figure 10a). By showing the 2TX-induced Fpg oxidation product, this crystal structure exquisitely supports the thiol/disulfide exchange mechanism we proposed above for the inhibition of ZnF-containing Fpg/Nei DNA glycosylases. With TXn, there was no clear electron density near the sulfur atoms of Cys248 and Cys268. According to the thiol/disulfide molecular mechanism (Figure 10b), this last observation suggests either the intermolecular disulfide bonds are too labile in the used crystallization conditions (spontaneous hydrolysis after oxidation) or the covalently linked TXn molecules are too dynamic to generate an interpretable electron density.

**Figure 11:**
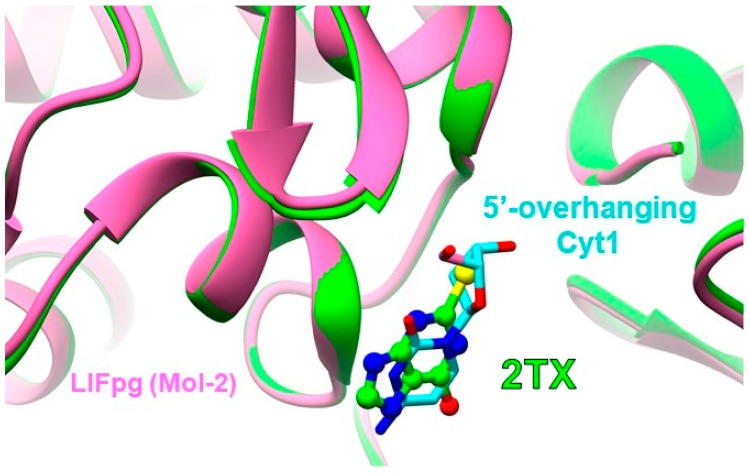
Superimposition of the crystal structure of LlFpg bound to an 8-oxoG-containing 14-mer DNA duplex (in *magenta*) with the crystal structure of LlFpg bound to a THF that contains the same DNA duplex in the presence of 2TX (in green) In this view, only the second LlFpg molecule bound to the extremity of the duplex (Mol-2) is visible (for an overview, see Figure S5, PDBid 4CIS). For clarity, DNA is voluntarily omitted. Only the 5′-overhanging cytosine (Cyt1) of the damaged strand in 4CIS is shown (in cyan).

Similar to the 2TX-containing crystal structure, the intramolecular disulfide bridge Cys245-S-S-Cys265 is also formed in the presence of TXn. Not observed previously, one 2TX molecule that is non-covalently bound to the enzyme is inserted at the protein-DNA interface in the vicinity of the ZnF loop and the H2TH motif (Figure 10a,b), the two DNA binding domains that characterize the Fpg/Nei DNA glycosylase superfamily [29]. This new 2TX binding site is included in site II proposed by the blind docking experiments described above (Figure 8). A similar non-covalent binding at site II is also observed with TXn (Figure 10c–g). Among the best docking poses observed with 2TX and TX19, some of them are very similar to the corresponding crystal structures (Figure S13). This last observation validates the docking procedure we used. The strictly conserved residues R260 of the ZnF loop and K57 of the β2-β3 loop of the N-terminal LlFpg domain are directly involved in the recognition of 2TX/TXn (Figure S11). The guanidinium group of the R260 side chain interacts with the unique sulfur atom of 2TX, TX15, TX20 and TX27, with the sulfur atom at the C8 position of TX19 (with or without TCEP) or with the N1 atom of the purine moiety of TX13. The epsilon amino group of the K57 side chain interacts with the sulfur atom of all 2TX/TXn compounds. R260 of ZnF of LlFpg (R277 of the ZnLF of hNeil1 and mvNei1) and K57 (K54 and K60 in hNeil1 and mvNei1, respectively) are key Fpg/Nei residues that are involved in DNA binding [36]. R260 and K57 are essential to neutralize the electronegativity of DNA phosphates that border the damaged nucleoside. They allow the enzyme to bend DNA. This DNA bending facilitates the extrusion of the damaged nucleoside outside the DNA helix and its stabilization inside the active site pocket that exposes it for catalysis [28,36,40]. In addition to its role in DNA binding, K57 is also proposed as an accessory catalytic residue for DNA glycosylase activity of the EcFpg [38,41,42]. Through binding at site II, the present crystal structures indicate that 2TX/TXn in their reduced forms do not disturb the local structure of the complex (the DNA backbone conformation and the side chain orientation of R260 and K57 are unchanged). In the absence of TCEP, we did not observe the fixation of the 2TX/TXn disulfide forms at sites I and II because they are probably consumed during oxidation of the protein ZnF and transformed into reduced species. This potential is consistent with the thiol/disulfide exchange mechanism we proposed (Figure 5b). For Fpg, the presence of TCEP in the incubation mixtures prevents ZnF oxidation by 2TX/TXn [18]. The crystal structure of Fpg bound to 14-mer [THF:C] in the presence of TX19 and TCEP revealed that the native structure of ZnF is preserved while one molecule of reduced form of TX19 is still observed at site II (Table S1). As anticipated by docking experiments on the enzyme/DNA complexes, the non-covalent binding site (docking site II) can be seen as a composite area partly formed by the protein and by the DNA backbone phosphates of the two-three nucleotides at the 3′ side of the damaged nucleotide (Figure 10b–g): the phosphate group of Thy(8) interacts with the sulfur group of TXn and the phosphate group of the nucleotide Thy(9) with the N1 atom of 2TX and TX13, the N7 atom of TX13, TX19 and TX27, the N9 atom of TX20, and the NH2 group substituted the C6 atom of the TX27 (Figure 10b). However, the binding of TX19_red_ does not alter the local DNA structure any more than that of the protein. Thus, it is very likely that the binding of the reduced 2TX/TXn species to site II should have little or no impact on Fpg/Nei protein activities and DNA binding. In the presence of TCEP, the disulfide forms of 2TX/TXn are reduced and are, thus, unable to inhibit the Fpg/Nei proteins (Figure 4). In addition, we suggest that the occupation of site II by some mono- or dithio-TXn or TXn without the thiol/thione group is incompatible with the fixation of a second Fpg molecule at the end of the DNA duplex (Figure 2a and Figure 3). Thus, the binding of such small molecules to site II may impair the formation of the C2 complex with or without TCEP observed in EMSA experiments with Fpg and in the crystal structure of LlFpg bound 8-oxoG-containing 14-mer DNA duplex (Figure 3, Figures S5 and S6). Indeed, fine inspection of the crystal structures of C2 [38] with that of the lesion recognition complex C1 resolved in the presence of 2TX, TX13, TX15, TX19, TX20 and TX27 showed that the 5′-overhanging cytosine of the damage-containing strand recognized by the second molecule (Mol-2) of LlFpg in C2 clashes with 2TX/TXn bound at the site II (Figure 11). This structural feature provides a satisfactory explanation to understand why some TXn (with or without thiol/thione group) in the presence of TCEP inhibit the formation of C2 (Figure 3 and Figure S6). At this stage of our investigations, we cannot exclude that the binding of the disulfide forms of monothio 2TX/TXn to site II contributes to the global inhibition of the ZnF-containing Fpg/Nei DNA glycosylases observed. As suggested by docking experiments, only the cyclic disulfide forms of the dithio species TX16 and TX19 might be responsible for the inhibition of the ZnLF-containing Fpg/Nei subclass enzymes (hNeil1 and mvNei1) by interacting more particularly with site III of the free enzyme. This proposal is relevant if site III identified by the docking experiments actually exists in hNeil1 and mvNei1. mvNei1, which is completely devoid of cysteine, is a good model to try to answer this question. Unfortunately, and despite numerous attempts, we did not succeed in crystallizing mvNei1 in the presence of the redox equilibrium mixtures of TX16 and TX19.

## 3. Materials and Methods

### 3.1. Chemicals and 2TX Derivatives Mini-Library

Sodium periodate (NaIO4) and TCEP were purchased from Sigma-Aldrich. The chemicals 2TX and 2-thio-6,8-dioxopurine (TX14) used to prepare the corresponding homo-disulfide compounds were supplied by MP Biomedicals and ChemCruz, respectively.

Design and synthesis procedures for the TXn used in this study are described in Supplementary Information. All molecule powders were dissolved at 4–10 mM final concentrations in a buffer containing 5 mM HEPES/NaOH (pH7.5), 80% DMSO, 40 mM KCl and 1% glycerol and stored for months at −20 °C.

### 3.2. Enzymes and DNA Probes

The wild type LlFpg was overproduced in the *fpg* defective *E. coli* strain BH540 and purified, as described previously [22,43]. The human hNeil1 nucleotide sequence was cloned in a pET30a+ expression vector (gift from Dr Susanne Wallace). The mimivirus Neil1 open reading frame (mvNei1) was codon optimized (Genscript) and cloned in pET22b+. The hNeil1 and mvNei1 proteins 6His-tagged at the C-terminus, were overproduced in *E. coli* Rosetta2 (DE3) strain in auto-inducible medium. The 6His-tagged proteins were purified by three liquid chromatography steps using HisTrap™ HP, HiTrap™ SP HP, and Hiload™ Superdex™ 75 pre-packed columns (GE Healthcare, Chicago, Illinois, USA). The hNeil1 and mvNei1 proteins were finally concentrated at 142 and 316 µM, respectively, in a buffer that contained 20 mM HEPES/NaOH (pH7.6), 500 mM NaCl, 1 mM TCEP and 10% glycerol. The truncated form of hNeil1 (∆hNeil1) was used for EMSA experiments. The ORF coding for ∆hNeil1 fused with a C-terminal 6His-tag was codon optimized (Genscript) and cloned in pET30a+. The recombinant pET30a+ was used to transform the BL21 (DE3) strain. Overproduction was performed as described above for full length hNeil1. The protein was purified as described above, concentrated at 195 µM in buffer that contained 20 mM HEPES/NaOH (pH 7.5), 150 mM NaCl, 1 mM DTT and 50% Glycerol. After purification, all proteins were stored at −80 °C after flash cooling in liquid nitrogen.

All synthetic oligodeoxyribonucleotides (ODNs) used in this study were purchased from Eurogentec. For catalytic and DNA binding activities (EMSA), the lesion-containing strand was labeled at its 5′ terminus with [γ^32^P]-ATP using T4 polynucleotide kinase. The 5′-[^32^P]-labeled 24-mer lesion-containing strand (5′-CTGATCGATGA**X**GCCTGACATGAT where X is for dihydrouracil (DHU) or 8-oxoguanine (8-oxoG)) was annealed with the cold complementary strand (5′-ATCATGTCAGGCYTCATCGATCAG where Y indicates C or G). The resulting DNA duplexes 24-mer 8-oxoG:C (OG:C) and 24-mer DHU:G (DHU:G) were used as substrates for measuring the catalytic activity of Fpg and hNeil1 (mvNei1), respectively. The 5′-[^32^P]-labeled 14-mer lesion-containing strand 5′-CTCTTTXTTTCTCG, where X is the tetrahydrofuran (THF) AP site analog, was annealed with the cold complementary strand (5′-GCGAGAAACAAAGA) to generate the 14-mer THF:C DNA duplex (THF:C) used for EMSA experiments (see below). The cold 14-mer THF:C DNA duplex was also used to crystallize Fpg/DNA complex with 2TX/TXn, as previously described [44].

### 3.3. Enzymes Assays for Inhibition and IC50 Determination

Fpg, hNeil1 and mvNei1 are bifunctional DNA glycosylases associated with concerted DNA glycosylase activity (cleavage of the *N*-glycosidic bond between the base lesion and the C1′ of the deoxyribose) and AP lyase activity that comprises the successive cleavage of the phosphodiester bonds at the 3′ and 5′ of the resulting abasic (AP) site, respectively (β, δ-elimination). Thus, AP sites that result from the excision of the damaged base are quantitatively converted into one nucleoside gap by these enzymes. The DNA glycosylase/AP lyase activity of each enzyme was measured by incubating 20 nM of 24-mer DNA duplexes ([OG:C] and [DHU:G] for Fpg and hNeil1/mvNei1, respectively) 5′-labeling on the damaged strand (see above) with 2 nM of Fpg, or mvNei1 for 15 min or, with 20 nM of hNeil1 for 30 min at 37 °C in 10 mM Tris-HCl, pH 7.6, 150 mM NaCl, 1 mM EDTA, 0.1 mg/mL BSA and 8% DMSO. The reaction was stopped by the addition of 1 volume of formamide loading buffer (96% formamide, 20 mM Tris/HCl pH 8.0 and 2 mM Na_2_EDTA), heated at 80 °C for 2 min and then analyzed by 20% Urea-PAGE, as previously described [45]. After electrophoresis, the gels were exposed for autoradiography, revealed using Typhoon imager and quantified with ImageQuant software. Under these conditions, only 50% of the substrate is converted to the cleavage product (limiting enzyme concentration conditions). For an initial evaluation of the inhibitory effect, 40 µM of 2TX/TXn was used. The thio-compound was added to the reaction mixture before the final addition of the enzyme (competition condition). For the best identified inhibitors, a dose-response curve was generated and IC50^A^ determined (Figure 1 and Table 1).

The DNA binding activity was measured using EMSA [37]. Briefly, 0.1 nM of the 5′-labeled 14-mer [THF:C] (see above) was incubated with the enzyme at 4 °C for 30 min in 25 mM HEPES/NaOH (pH 7.5), 150 mM NaCl, 5% glycerol, 0.1 mg/mL BSA and 8% DMSO. The incubation mixture was then loaded onto a native 10% polyacrylamide gel (Acryl/Bis 19/1) and electrophoresed at 25 V/cm for 90 min at 4 °C. After electrophoresis, the gels were dried and exposed overnight for autoradiography using a Typhoon imager and quantified with ImageQuant software. To evaluate the inhibitor effect of 2TX/TXn on the DNA binding activity of each Fpg/Nei enzyme, we first determined the experimental conditions required to bind 50% of the DNA probes without an inhibitor in the presence of 8% DMSO (limiting enzyme concentration). Under these conditions, we obtained the half-maximal DNA binding 0.25, 7 and 5 nM of Fpg, hNeil1 and mvNei1. We used this limited enzyme concentrations to evaluate the inhibitor effect of 2TX/TXn on the enzyme DNA binding activity. This effect was determined by the addition of the thio-compound in the pre-equilibrium mixture of the enzyme and DNA probe (30 min) and incubated for an additional 30 min at 4 °C. For the best inhibitors of Fpg, a dose-response curve was established from at least three independent experiments and the IC50 was determined (IC50^B^, Table 1).

### 3.4. Molecular Dynamic Simulation and Docking

Four 3D structures were used as receptors for ligand docking: (i) the 3D model of free Fpg from *L. lactis* (LlFpg) obtained by removing DNA duplex from the X-ray structure of the enzyme bound to THF-containing 14-mer DNA duplex, (ii) the X-ray structure of LlFpg/DNA-THF complex (PDBidcode: 1PM5), (iii) the X-ray structure of free hNeil1 (PDBidcode: 1TDH) for which missing residues were reconstructed using the Prime module from Schrödinger (Schrödinger, LLC, New York, NY, 2017) (https://www.schrodinger.com/) and (iv) the X-ray structure of hNeil1 bound to THF-DNA (PDBidcode: 5ITT).

The four systems were placed in a rectangular box with explicit TIP3P water molecules with a 10 Å buffer. To ensure the neutrality of the systems, counter ions were added. The systems were then energy minimized. Molecular dynamics (MD) simulations of 50 ps were then performed during which the temperature was increased in 50 K steps to reach the target temperature of 310 K. Equilibration were performed in seven MD steps of 50 ps, with positional restraints on the heavy atoms that incrementally decreased from 100 to 0.0 kcal/(mol.Å^2^). For each system, a 40 ns MD production run was performed in the NPT ensemble and conformations were stored every 1 ps for analysis. For energy minimization and MD simulations SHAKE algorithm [46] was used with a 2 fs time step for integration of the equation of motion. The particle mesh Ewald summation [47] was used for electrostatics calculation with a 12 Å cutoff. Periodic boundary conditions were applied. For MD simulation, the Amber package (Amber 11) with the ff12SB force field was used (https://ambermd.org/).

For the two systems containing a THF (tetrahydrofuran), the geometry and charge distribution of the THF residue were computed using Gaussian 03 program (https://gaussian.com/) and fitted with the RESP program [48] for compatibility with Cornell et al.’s force field [49] adapted to Parm98 parameters. For each system, a Hierarchical Ascending Clustering (HAC) method by average linkage distance calculation was used to classify system conformations in clusters based on a 2D RMSD map (each configuration of a trajectory was compared to all the others). We extracted one structure, called centroid, for each cluster. A centroid is representative of the system flexibility within a single cluster. The thirty collected centroids were then used for docking studies.

Reduced and disulfide forms of 2TX were sketched within the Sybyl suite and their 3D structures calculated with Concord and their energy minimized. For the disulfide dimer of 2TX, several conformational search techniques (Random search, GA search and Confort with the default parameters set in Sybyl package) were used to have a representative set of conformers. To obtain an exhaustive (as possible) set of cyclic conformers for docking with TX19_Ox1,Ox2_, we used several methods such as Random search, Grid search and simulated annealing, as implemented in the Sybyl package (Certara, NJ, USA), all with default parameters. To prepare the docking with Surflex-Dock 2.706 Surflex Manual: Docking and Similarity Version 2.7 (May 2012) by BioPharmics LLC (Santa Fe, CA, USA), a protomol was generated for each protein structure (centroids) in automatic mode with default parameters from the Sybyl interface. Protomols can be seen as an ‘imprinting’ an interaction cavity of a protein. The docking studies were performed in GeomX mode; the other options were set as -spindense 9.0, +premin, +remin, -multistart 6, -div_rms 0.5, -ndock_final 20 and +ring. For each pre-calculated conformer, Surflex-Dock will generate 6 subsequent conformers prior to the docking. Twenty poses are proposed at the end.

### 3.5. Crystallization and X-Ray Structure Determination

LlFpg/DNA complexes (70–160 µM) were prepared by mixing in a 1.5 molar excess the 14-mer [THF:C] DNA duplex with LlFpg and crystallized at room temperature in the presence of 0.5% DMSO and with (for TX19) or absence of TCEP using a previously described method [18,22,23]. Crystals with 2TX, TX13, TX15, TX19, TX19 (+TCEP), TX20 or TX27 were obtained by soaking crystals of LlFpg/DNA complex in 2TX (TXn)-saturated mother liquor. Soaked crystals were then flash cooled in liquid nitrogen. X-ray diffraction data sets were collected at 100 K at beamline PROXIMA-1 and PROXIMA-2 (SOLEIL, Paris) or ID30A-3/MASSIF-3 (ESRF, Grenoble). All data were processed using the XDS package [50] and the CCP4 program SCALA [51]. The 3D structures were solved by molecular replacement using the crystal structure of the wild type LlFpg protein bound to 14-mer [THF:C] (PDB ID code 1PM5, [35]) as previously described [18]. X-ray data collection and refinement statistics are presented in Table S3. All structure cartoons were generated with CCP4MG [52].

## 4. Conclusions

DNA glycosylases, which initiate base excision repair (BER), constitute the first line of defense against oxidative stress resulting in nucleobase oxidation. Paradoxically, the very high level of oxidative stress in viral and bacterial infections, in cancer cells and in the cells of patients suffering from inflammatory diseases, such as Huntington’s disease, give reason to hope that the DNA glycosylases hOgg1 and hNeil1 are relevant pharmacological targets in precise pathologic situations [13,15,17,53,54,55,56]. Although many DNA repair enzymes, such as PARP1, are considered as therapeutic targets [57], the advantage of selectively targeting DNA glycosylases in cancer and neurodegenerative diseases has emerged only recently. The redundancy of DNA glycosylase substrate specificity and the observation that single KO mouse of hNeil1 and hOgg1 are viable and fertile have long hampered the search for inhibitors of these enzymes. Yet this situation is ideal in the case of a synthetic lethality interaction between the pathologic context and DNA glycosylase [58,59]. Over the last few years, the search for inhibitors of DNA glycosylases hNeil1 (hNeil2) and hOgg1 has become a very active field [18,19,20,21,56,60,61]. We started this research in 2014 by discovering that 2-thioxanthine is an irreversible inhibitor of the bacterial enzymes Fpg and Nei and the human enzyme hNeil2, three enzymes belonging to the Fpg/Nei DNA glycosylase structural superfamily having a zinc finger (ZnF) [18]. Interestingly, only hNeil1 from this enzyme superfamily is resistant to the inhibitory effect of 2TX. We clearly demonstrated that 2TX is able to irreversibly oxidize the Fpg ZnF. Although this inhibition mechanism remains to be elucidated, this finding provided an explanation permitting to understand why hNeil1 is resistant to 2TX knowing that this enzyme does not have ZnF.

In the present work, we described the discovery and biochemical characterization of new structural Fpg and hNeil1/mvNei1 inhibitors. By screening a small library of 2TX derivatives (TXn), the initial goal of the study was to find inhibitors that were more effective than 2TX and to decipher the mode of action of these thio-compounds on the Fpg/Nei DNA glycosylases. Four TXn (mono- or dithio-thiopurines) are better Fpg inhibitors than 2TX. Especially, 2-trifluoro-6-thiopurine (TX13) is seven times more effective than 2TX. As anticipated by our previous study, most TXn tested are unable to inhibit hNeil1 and mvNei1. What was not expected at the beginning of this study, however, is that TX16 and TX19, the two dithiopurines evaluated, also efficiently inhibit hNeil1 and mvNei1, DNA glycosylases devoid of ZnF. Knowing that mvNei1 is free of cysteine, its inhibition by TX16 and TX19 seemed very mysterious. Even more curious, TCEP, a very powerful reducer of disulfide bridges, annihilates the inhibitor effect of TX16 and TX19 on hNeil1 and mvNei1 glycosylase activity and DNA binding. A similar effect was observed for the action of TCEP in the presence of monothio-compounds. This suggested that TCEP acts directly on 2TX/TXn.

By combining mass spectrometry, chemistry, biochemistry and X-ray structure analysis, we demonstrated that only the disulfide forms of 2TX, TX14 and TX19 (in equilibrium with the reduced form in our DMSO mixtures of 2TX/TXn) are responsible for the inhibition of the Fpg/Nei enzymes. Although reduced 2TX/TXn are able to bind the free and DNA bound enzymes, as proposed by blind docking and confirmed by X-ray crystal structures of ternary complexes Fpg-DNA-2TX/TXn, their binding either in site II or in the active site (site I) observed with or without TCEP is probably not associated with inhibition. Disulfides are known to inhibit several enzymes according to various mechanisms. Unsymmetrical aromatic disulfides obtained from monothiolated compounds inhibit the SARS-CoV M^pro^ (an important target to fight severe acute respiratory syndrome-associated coronavirus, SARS-CoV) probably by interacting with the catalytic cysteine containing the protease M^pro^ [62]. Li et al. reported the synthesis of unsymmetrical aromatic disulfides as novel inhibitors (“herbicides”) of the acetohydroxyacid synthase, a plant and microorganism enzyme involved in the biosynthesis of branched-chain amino acids [63]. Reynolds and co-workers published unsymmetrical aryl-alkyl disulfides that selectively inhibit the methicillin-resistant *Staphylococcus aureus* and *Bacillus anthracis* [64]. The use of disulfides to selectively target the human ZnF- and ZnLF-containing Fpg/Nei DNA glycosylases (hNeil1, hNeil2 and hNeil3) in therapy is, however, conditioned to their activity in plasma (antioxidant power mediated by human serum albumin, HSA; [65]) and in the presence in cells of reduced glutathione (G-SH), which remains to be determined. Taking into account the potential reduction of the disulfide inhibitors by HSA, all enzyme assays presented in this work were performed in the presence of BSA (the bovine functional homolog of HSA). Regarding G-SH, the mixed disulfide Glutathione G-S-S-compounds retain in vivo activity [66]. In pathologic contexts associated with high levels of oxidative stress, the antioxidant power of G-SH and HSA can be greatly reduced and, thus, are incapable of countering the action of disulfides. Some aromatic disulfides were shown to be active in vivo as antitumor agents [67].

In the case of ZnF-containing Fpg/Nei enzymes, we demonstrated that ZnF oxidation results from a thiol/disulfide exchange mechanism between cysteine residues coordinating the Zn^2+^ ion and the 2TX/TXn disulfides. Thus, these disulfides act as Zn ejectors, which results, in our case, in an irreversible inhibition. Such a mechanism has already been proposed for the inhibition of metallo-β-lactamases by symmetrical disulfides, which induced the selective oxidation of the Zn(II)-coordinated cysteine center in the active site [68]. The natural symmetrical disulfide Psammaplin A isolated from sponge, and its analogs were described as antitumor agents by strongly inhibiting DNA methyltransferase (DNMT) and histone deacethylase (HDAC) interfering with their respective Zn center [67]. A thiol/disulfide exchange mechanism resulting in ZnF oxidation and Zn release has also been proposed for the inhibition of the HIV-1 nucleocapside protein by the Zn ejector DIBA (a symmetrical disulfide) [69]. Blind and flexible docking also suggests that inhibition partly results from the binding of the disulfide to the active site (site I) and/or site II.

The most striking result of this study is the discovery that disulfide forms of dithio-TXn (TX16 and TX19) are also capable of inhibiting ZnLF-containing Fpg/Nei DNA glycosylases like hNeil1 and mvNei1. For TX19, we showed that the thio-compound exists in DMSO solution in equilibrium between TX19_Red_ and its two cyclic trimeric disulfide forms TX19_Ox1,Ox2_. Through binding at sites I and II, EMSA experiments and blind and flexible docking suggest that the inhibitory effect of TX19_Ox_ could be effective on both the free and DNA bound hNeil1 and mvNei1. Docking of TX19 disulfides revealed a third binding site (site III) only accessible on the free Fpg and hNeil1. If this site really exists, the binding of Fpg and hNeil1 to DNA could be inhibited when a disulfide of TX19(TX16) occupies site III. The self-assembly property of the aromatic dithio-compounds, such as TX16 and TX19 in cyclic disulfides (disulfide cyclophanes), has been known for a long time by chemists. Thus, symmetrical and unsymmetrical disulfides are useful tools for creating small molecule libraries by dynamic combinatorial chemistry (DCLs) [70]. More recently, this method was adapted to search for enzyme inhibitors using the protein as a molecular trap (named “protein-directed combinatorial chemistry”, PDCC) [71]. Combined with a fragment-based screening method, PDCC is an alternative to the expensive high-throughput screening (HTS) of chemical libraries and enables the exploration of a larger chemical space in a more efficient manner. Our work modestly illustrates the interest of this strategy to find new enzyme inhibitors and potential protein target sites that can inspire the design of new classes of inhibitors of hNeil1. The discovery that disulfide cyclophanes inhibit Fpg and hNeil1 (mvNei1) constitutes the starting point in the exploration of new avenues to find new potent inhibitors with original structures using PDCC combined with fragment-base screening. We are in the process of implementing this strategy for hNeil1 and hOgg1. 

## Figures and Tables

**Table 1:** Effect of TXn on Fpg/Nei catalytic and DNA binding activities.

Compound		IC50_app_^A^ (µM) (Fpg/Nei enzyme catalysis)			IC50_app_^B^ (µM) (Fpg DNA binding) *	
		*LlFpg*	*hNEIL1*	*mvNEI1*	*C1*	*C2*
2TX *	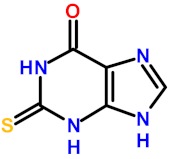	48 ± 4	>500	>500	113.3 ± 17 (453)	NA (9.7)
TX13 *	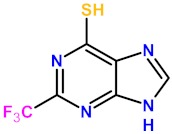	7.5 ± 1.0	>250	>500	6.1 ± *0.5* (98)	NA (1.3)
TX14 *	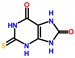	28 ± 7	>500	>500	25.1±2.4 (100)	NA (2.8)
TX16 **	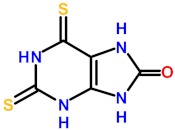	41 ± 7	21 ± 1	14 ± 1	ND	NA
TX19 **	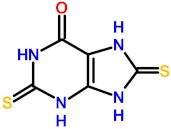	15 ± 1	19 ± 1	124 ± 4	17.1 ± 2.4 (68)	NA (2)
TX27 *	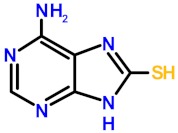	20 ± 2	160 ± 16	277 ± 18	44.19 ± 8.0 (177)	NA (4)

IC50_app_ corresponds to the half-maximal inhibitory concentration of a compound with (^A^) and (^B^) being relative to the catalytic activity at 37 °C and the DNA binding activity at 4 °C, respectively. ND and NA indicate not-determined and not-applicable, respectively. * Determined with 0.1 nM of 14-mer [THF:C] DNA duplex and 0.25 and 10 nM of Fpg for C1 and C2, respectively. C1 corresponds to the specific lesion recognition complex (lesion recognition complex (LRC), Fpg/DNA = 1/1), and C2 corresponds to the non-specific binding of a second Fpg molecule to the preformed C1 (Fpg/DNA = 2/1; see text below). The numbers in parentheses indicate the number of TXn equivalents to be added to one protein equivalent to observe 50% inhibition of DNA binding activity.

## Supplementary Materials

Supplementary materials can be found at https://www.mdpi.com/1422-0067/21/6/2058/s1.

## Abbreviations

EMSA	Electrophoretic mobility shift assay
2TX	2-thioxanthine
TXn	2TX derivatives
LRC	Lesion recognition complex
BER	Base excision repair
LCL	Lesion capping loop
DMSO	Dimethylsulfoxide
THF	Tetrahydrofuran
TCEP	Tris(2-carboxyethyl)phosphine hydrochloride
DTT	Dithiothreitol
8-oxoG	8-oxoguanine
AP site	Abasic site
ZnF	Zinc Finger
ZnLF	Zincless Finger
