# Supplementary material for: Thiopurine Derivative-Induced Fpg/Nei DNA Glycosylase Inhibition: Structural, Dynamic and Functional Insights
Source: Int J Mol Sci. 2020 Mar 17;21(6):2058. doi: 10.3390/ijms21062058 (PMC7139703; doi:10.3390/ijms21062058)
Supplement: Supplementary file 1 [file ijms-21-02058-s001.pdf]

SUPPLEMENTARY INFORMATION

Thiopurine derivative-induced Fpg/Nei DNA glycosylase inhibition: structural, dynamic and functional insights

Charlotte Rieux[1,2], Stéphane Goffinont[1], Franck Coste[1], Zahira Tber[2], Julien Cros[1], Vincent Roy[2,3], Martine Guérin[1,3], Julien Cros[1], Virginie Gaudon[1], Stéphane Bourg[2], Artur Biela[1], Vincent Aucagne[1], Luigi Agrofoglio[2,3], Norbert Garnier[1,3] and Bertrand Castaing[1]

(1) Centre de Biophysique Moléculaire, UPR4301 CNRS, rue Charles Sadron, F-45071 Orléans Cédex 2, France
(2) Institut de Chimie Organique et Analytique, UMR7311 CNRS-Orleans University, Université d'Orléans, Pôle de Chimie, rue de Chartres, F-45100 Orléans, France
(3) Université d'Orléans, UFR Sciences et Techniques, rue de Chartres, 45100, Orléans, France

• Chemical synthesis strategies for the design of the small 2-thioxanthine derivative (TXn) library

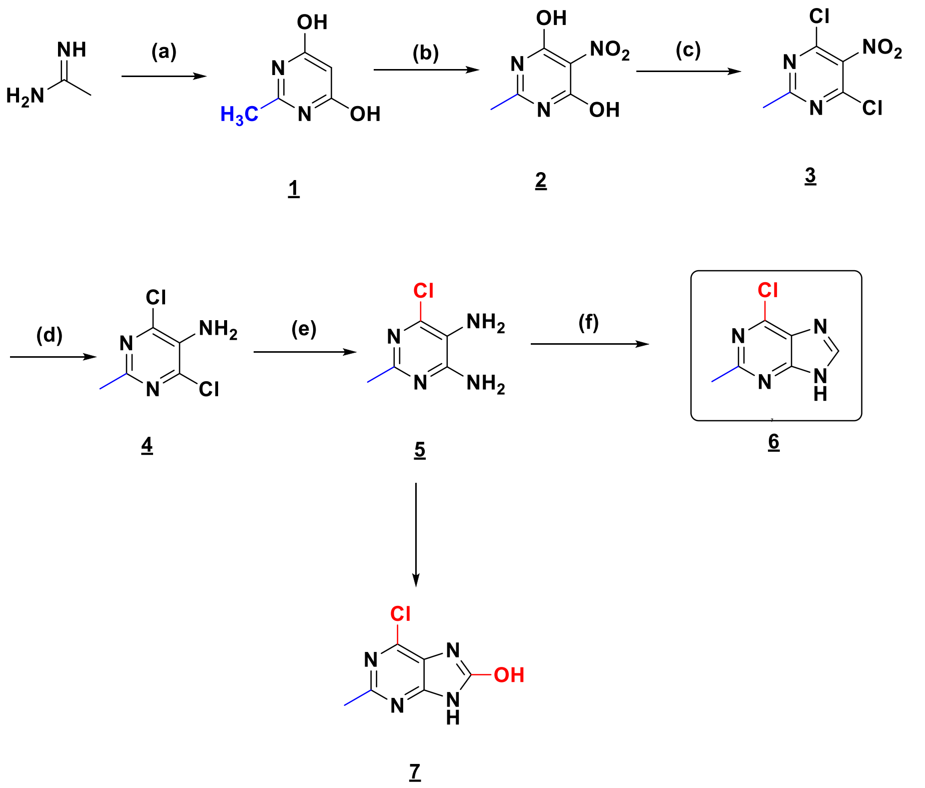

(a) EtONa, diethylmalonate, 85°C, 3 h, 69%; (b) HNO_3 fumant, acetic acid, 0°C, 3 h, 67%; (c) $POCl_3$, *N*,*N* diethylaniline, 115°C , 2h 30, 64%; (d) Fe , AcOH, 65°C, 82%; (e) NH_4OH, 120°C, 25 min, Mw, 70%; (f) Triethylorthoformate, 75 min, 100°C, 84%; (g) Urea, 160°C, 1h, 20%.

<u>**Scheme 1**</u>

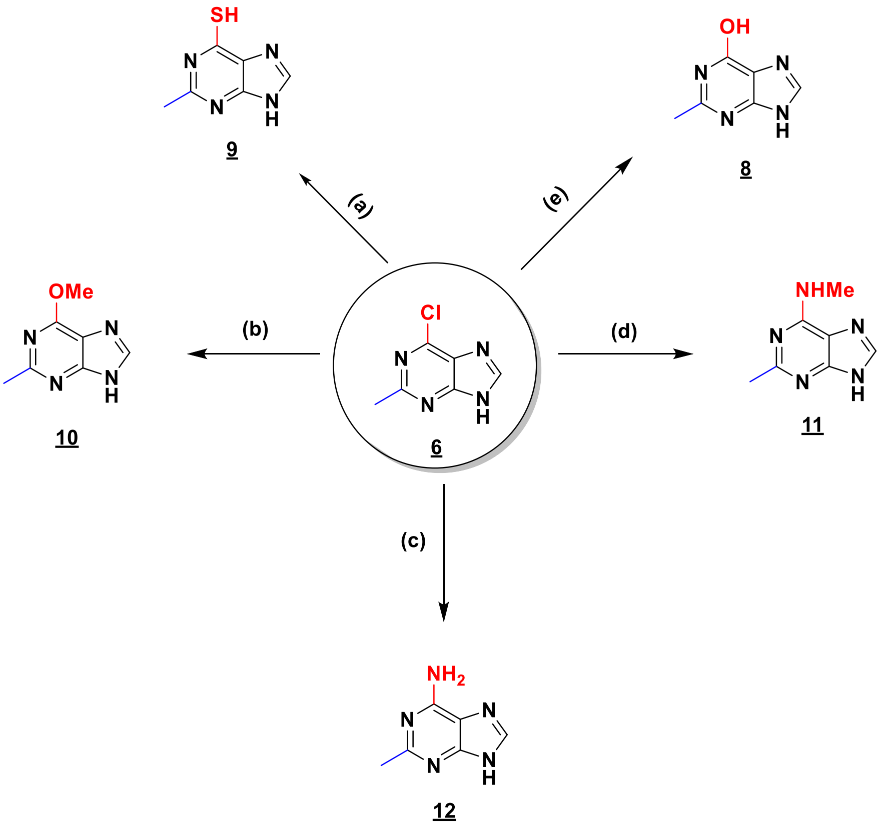

(a) Thiourea, EtOH, reflux, 1h, 35%; (b) NaOH, MeOH, Mw, 100°C, 4min, 81%; (c) 1) NaN_3, EtOH/H_20, 80°C , 2 h, 2) $SnCl_2$,$2H_20$, reflux, 2 h, 74%; (d) $MeNH_2$, EtN_3, BuOH, 140°C, Mw, 15 min, 58%; (e) HCOOH/H_2O (1/1), 40°C , 20 h, 80%.

Scheme 2

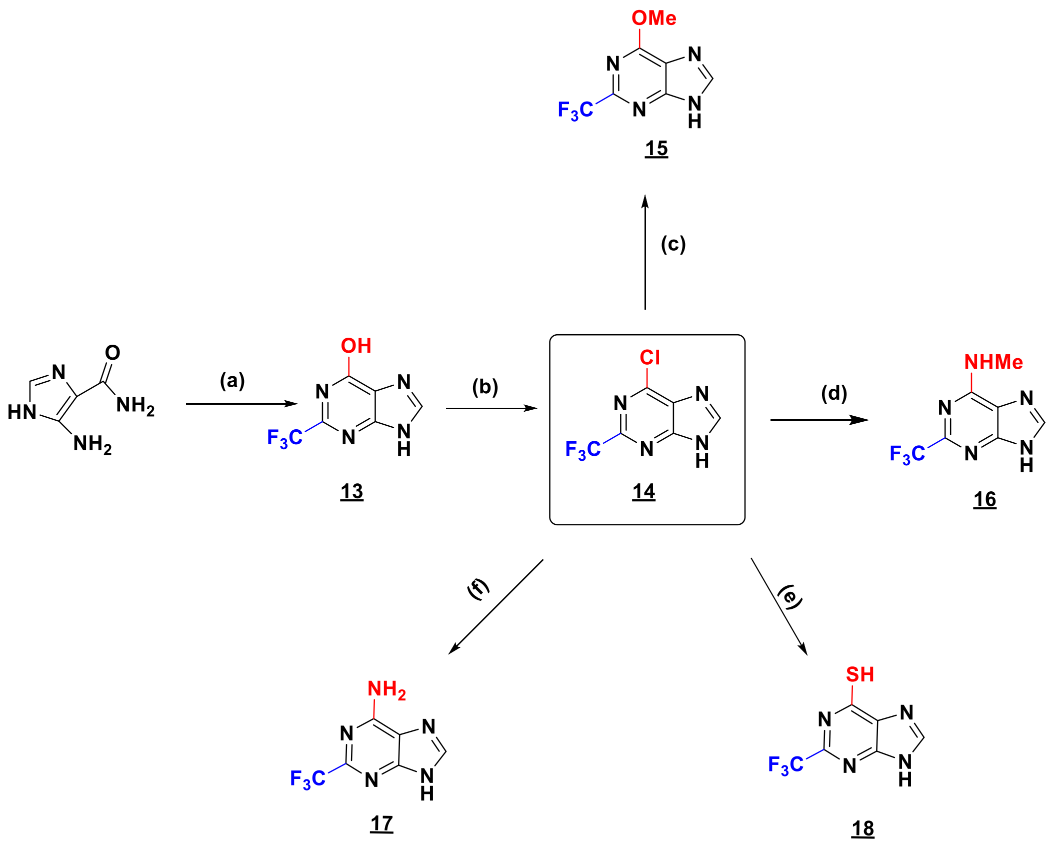

(a) trifluoroacétamide, 160°C, 4 h, 48%; (b) $SOCl_2$, DMF, $CHCl_3$, reflux, 64%; (c) NaOH, MeOH, Mw, 100 °C, 40 min, 79%;(d) $MeNH_2$,BuOH, Et_3N, Mw, 140°C, 15 min, 53%; (e)Thiourea, EtOH, reflux, 4h, 54%; (f) 1) NaN_3 , EtOH/H_2O, 2h , 80 °C, 2) $SnCl_2$,$2H_2O$, EtOH, 2 h, reflux, 67%

Scheme 3

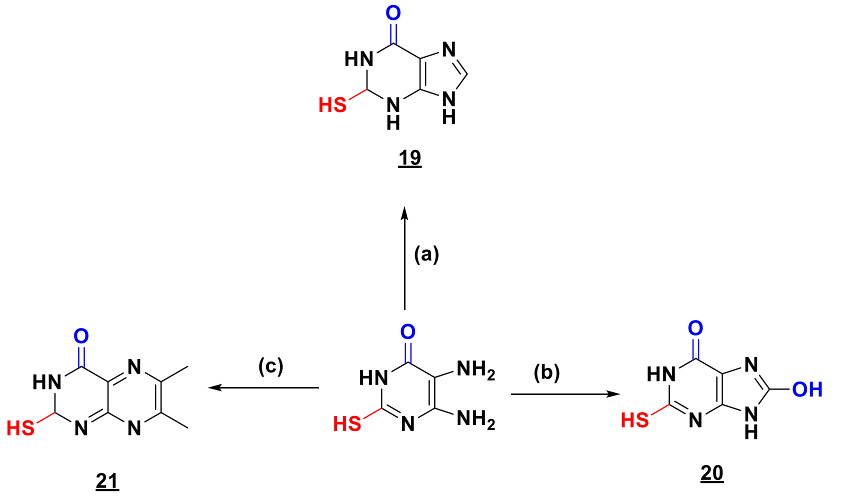

(a) 1) formic acid, 2 h, reflux; cooled, 2) H_2NCHO, 2 h, 175-185°C, 97%; (b) Urea, 170°C, 30 min, 90% ; (c) Diacetyl, DMF, 100°C, 5h, 67%.

Scheme 4

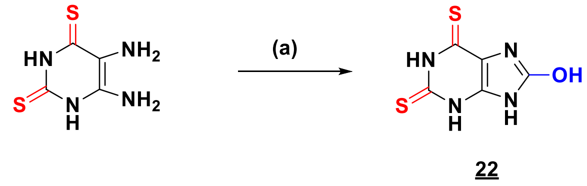

(a) Urea, 170°C, 45 min, 60%

Scheme 5

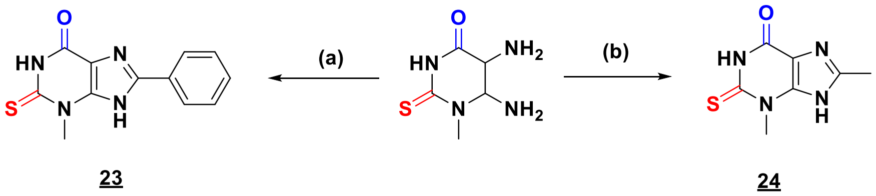

(a) triethoxymethylbenzene, reflux, 15h, 90%; (b) triethylorthoacetate,reflux, 15h, 60%

Scheme 6

The reactions were monitored by thin-layer chromatography (TLC) analysis using silica gel (60 F254) plates. The compounds were visualized by UV irradiation. Flash column chromatography was performed on silica gel 60 (230−400.13 mesh, 0.040 0.063 mm). The infrared spectra of compounds are given in cm^{-1}. Further, 1 H and 13C NMR spectra were recorded at 250 nm (13C, 62.9 MHz) or at 400 nm (13C, 100.62 MHz). Chemical shifts are given in parts per million using tetramethylsilane (TMS) as an internal standard. Coupling constants (J) are reported in Hertz (Hz). All reactions under microwave irradiation were performed using the Biotage Initiator microwave in 2-5 mL or 10-20 mL sealed tubes. High-resolution mass spectra (HRMS) were performed on a quadrupole analyzer.

4,6-Dihydroxy-2-methylpyrimidine <u>1</u> (CAS : 1194-22-5)

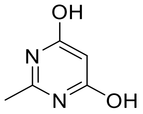

To a stirred solution of sodium ethoxide, prepared from sodium (210 mg, 9.13 mmol) and ethanol (4 mL), acetamidine hydrochloride (290 mg, 3.066 mmol, 1 eq) was added in one portion (rinsed with ethanol, 0.5 mL). After 5 min, diethyl malonate (0.41 mL, 2.7 mmol, 0.9 eq) was added in the above suspension (rinsed with ethanol, 1 mL). The reaction mixture was refluxed (85°C) for 3 h, allowed to cool to room temperature, and diluted with water (4 mL). After all the precipitate was dissolved, concentrated hydrochloric acid (0.6 mL) was added dropwise. The precipitate was collected, washed with water (2 mL), ethanol (2 mL) and ether (2 mL), and then dried under a vacuum.

White solid (69%); **IR:** 3078, 2793, 2584, 1622, 1573, 1450, 1325, 1041, 982, 933; **^{1}H-NMR (DMSO-D_6, 250 MHz):** δ 4.94 (s, 1 H, H-5), 3.33 (s, 2 H), 2.20 (s, 3 H, CH_3); **HRMS (ESI): m/z (M+H)** $^{+}$ calculated for $C_5H_7N_2O_2$: 127.05020; found: 127.05019.

2-Methyl-5-nitro-4,6-dihydroxypyrimidine <u>2</u> (CAS : 53925-27-2)

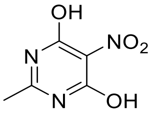

To a mixture of nitric acid (fuming, 0.5 mL, 6 eq) and acetic acid (glacial, 0.25 mL) at 10–15°C, <u>1</u> (246 mg, 1.86 mmol, 1 eq) was added in portions over a period of 30 min. The reaction mixture was stirred for 3 h at room temperature, cooled to 0°C, diluted with cold water (0–5°C, 0.4 ml), stirred for 3 min more, and then filtered. The solid was washed with cold water (0–5°C, 0.2 ml), ethanol (1 mL) and ether (2 mL) and dried under a vacuum.

White solid (67%); **IR**: 3537, 3458, 2815, 2711, 1673, 1627, 1361, 1299, 1275, 1050, 785; **^{1} H-NMR (DMSO-D_6, 250 MHz):** δ 12.98 (brs, 2H), 2.31 (s, 3H, CH_3); **HRMS (ESI): m/z (M+H)** $^{+}$ calculated for $C_5H_6N_3O_4$: 154.02471 found : 154.02476

2-Methyl-5-nitro-4,6-dichloropyrimidine <u>3</u> (CAS : 13162-43-1)

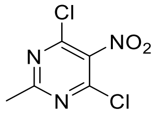

To a suspension of **<u>2</u>** (241 mg, 1.36 mmol, 1 eq) in phosphorus oxychloride (1 mL) *N,N*diethylaniline (0.28 mL, 1.76 mmol) was added dropwise. The reaction mixture was refluxed (115°C bath) for 2.5 h and then cooled to room temperature. Excess phosphorus oxychloride was evaporated under a vacuum. The residue was diluted with ether and poured onto ice. The water layer was extracted with ether (3x5 mL). The combined organic extract was washed with saturated solutions of sodium bicarbonate and sodium chloride, dried over sodium sulfate, and evaporated under a vacuum.

Light brown crystals (64 %); **IR**: 3537, 3464, 2923, 2847, 2701, 1673, 1627, 1589, 1360, 1280, 1048, 784; **^{1}H NMR (DMSO-D$_6$, 250 MHz):** δ 2.7 (s, 3H); **HRMS (ESI) :** not detected.

5-Amino-4,6-dichloro-2-methylpyrimidine <u>4</u> (CAS : 39906-04-2)

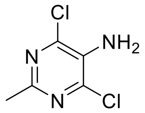

The product **<u>3</u>** (20.2 g, 0.097 mol, 1 eq) was dissolved in a mixture of hydrochloric acid (10 mL) and ethanol (200 mL). One portion of iron powder (16.4 g, 0.293 mol, 3 eq) was added to this solution. The mixture was then refluxed for 8 h, cooled to room temperature, and filtered through a pad of Celite. The filtrate was concentrated in vacuo. The residue was extracted with EtOAc, and the organic extract was washed with 1 N NaOH, water and brine and dried over anhydrous $MgSO_4$. It was then filtered and concentrated in vacuo to a tan solid. Purification by recrystallization from water yielded the pure product.

White crystals (82 %); **IR**: 3449, 3336, 3341, 3293, 1607, 1505, 1430, 1315, 1123, 913, 788; **^{1}H NMR (DMSO-D$_6$, 250 MHz):** δ 5.88 (brs, 2H), 2.40 (s, 3H); **HRMS (ESI): m/z (M+H)$^+$** calculated for $C_5H_6N_3Cl_2$: 177.99332 found : 177.99323

2-Methyl-4,6-dichloro-5-aminopyrimidine <u>5</u> (CAS : 933-80-2)

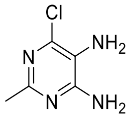

The product **<u>4</u>** (1.00 g) and ammonium hydroxide (5.0 mL, 28.0% - 30.0 %) were placed in a microwave vial. The vial was sealed and heated in a microwave reactor at 120 °C for 25 min. The reaction mixture was cooled to room temperature and concentrated to give 6-chloro-2-methylpyrimidine-4,5-diamine.

Light brown solid (70 %); **IR :** 3347, 3293, 3131, 3034, 2800, 1657, 1545, 1397, 1236, 865; **^{1}H NMR (DMSO-D$_6$, 250 MHz)** δ 6.65 (s, 2H, NH_2), 4.71 (s, 2H, NH_2), 2.18 (s, 3H, CH_3)**; HRMS (ESI): m/z (M+H)$^+$** calculated for $C_5H_8N_4Cl$: 159.04320 found : 159.04311

2-Methyl-6-chloropurine 6 (CAS : 100859-35-6)**: TX1**

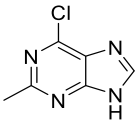

The compound 5 (0.5 g, 3.155 mmol, 1 eq) was suspended in triethyl orthoformate (5.62 mL, 33.8 mmol, 10.7 eq), placed in a preheated oil bath (100 °C) and stirred for 75 min. The reaction mixture was cooled to room temperature, concentrated, treated with hexanes and filtered. The solid was washed with hexanes and dried to yield 6-chloro-2-methyl-9H-purine.

Light brown solid (84%); **IR:** 3110, 2955, 2923, 2768, 2714, 2673, 1613, 1592, 1386, 1292, 1224, 952, 886, 810; **^{1}H NMR (DMSO-D$_6$, 250 MHz)**: δ 13.7 (brs, 1 H, NH), 8.55 (s, 1H, H-8), 2.65 (s, 3 H, CH$_3$)**; HRMS (ESI) : m/z (M+H)**$^+$ calculated for $C_6H_6N_4Cl$: 169.02755 found : 169.02747

6-chloro-1,7-dihydro-2-methyl- 8*H*-Purin-8-one 7 (CAS: 98138-74-0)**: TX2**

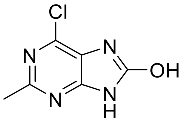

The compound 5 (0.100 g, 0.63 mmol, 1 eq) and urea (0.213g, 3.5 mmol, 5.5 eq) are mixed in a flask with stirring and heated to 160 ° C for 45 min. The agitation became difficult. The residue was purified by silica gel column chromatography.

White solid (20%); **IR**: 3129, 3021, 2980, 2777, 1738, 1593, 1426, 1392, 1222, 1182, 1043, 1005, 901, 711, **^{1}H NMR (MeOH-D$_4$, 250 MHz):** δ 2.79 (s, 3H, CH$_3$)**; HRMS (ESI): m/z (M+H)**$^+$ calcd for $C_6H_6N_4ClO$: 185.02246 found : 185.02213

2-Methylhypoxanthine 8 (CAS : 5167-18-0)**: TX3**

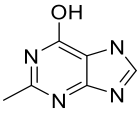

A solution of 6 (100 mg, 5.94 mmol, 1 eq) in a 1:1 mixture of water (8.7mL) and formic acid (8.7 mL) was stirred for 20 h at 40 °C. After evaporation of all volatiles, the residue was purified by silica gel column chromatography (EtOAc/MeOH, 9:1)

White solid (80%); **IR** 3092, 3027, 2927, 2797, 2679, 1686, 1364, 1260, 1185, 957, 807; **^{1}H NMR (DMSO-D$_6$, 250 MHz):** δ 13.10 (brs, 1 H, NH), 12.09 (brs, 1 H, OH), 8.12 (s, 1H, H-8), 2.35 (s, 3H, CH$_3$), **HRMS (ESI): m/z (M+H)**$^+$ calculated for $C_6H_7N_4O$: 151.06143 found : 151.061224.

2-Methyl-6-mercaptopurine 9 (CAS: 38917-31-6)**: TX4**

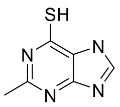

A solution of **6** (200 mg, 1.19 mmol, 1 eq) and thiourea (399 mg, 5.24 mmol, 4.42 eq) in ethanol was stirred at reflux for 1h. After cooling to room temperature, the result was filtered, and the residue was purified by silica gel column chromatography (EtOAc/MeOH, 8:2)

Beige solid (35%), **IR:** 3010, 2946, 25581551, 1418, 1356,1191, 1137, 1007, 794, **^{1}H NMR (DMSO-D$_6$, 250 MHz)**: δ 8.61 (s, 3H), 2.70 (s, 1H), **HRMS (ESI): m/z (M+H)** $^{+}$ calculated for $C_6H_7N_4S$: 167.038594 , found : 167.038676

6-methoxy-2-methyl-9*H*-Purine 10 (CAS: 1198-45-4)**: TX5**

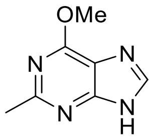

A 0.5-2 mL vial was loaded a solution of **6** (0.1 g, 0.593 mmol, 1 eq) and NaOH (71 mg, 1.77 mmol, 3 eq) in methanol (1.2 mL). After microwaving at 100°C for 40 min, the volatiles were evaporated under reduced pressure. The resulting residue was then purified by silica gel chromatography (EtOAc/ MeOH).

White solid (81%) ; **IR :** 3008, 2945, 2752, 2546, 1613, 1591, 1566, 1397, 1382, 1268, 1116, 949, 794, **^{1}H NMR (DMSO-D$_6$, 250 MHz)**: δ 13.16 (brs, 1H, NH), 8.25 (s, 1 H, H-8), 4.05 (s, 3H, CH_3), 2.52 (s, 3H, CH_3), **HRMS (ESI): m/z (M+H)** $^{+}$ calculated for $C_7H_9N_4O$: 165.077087 found : 165.077014

2-Methyl-6-methylaminopurine 11 (CAS : 90375-79-4)**: TX6**

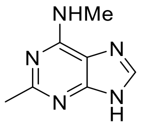

A 2-5 mL vial was charged with **6** (0.150 g, 0.88 mmol, 1 eq), *n*-butanol (1.81 mL), methylamine (0.21mL, 5.33 mmol, 6 eq), and distilled triethylamine (0.37 mL, 2.6 mmol, 3 eq). After microwaving at 140 °C for 15 min, the solvent was removed under pressure, and the residue was purified by silica gel column chromatography (EtOAc/ MeOH).

White solid (58 %); **IR:** 3274, 3208, 3059, 2983, 2942, 1606, 1384, 1330, 1264, 1137, 931, 825, 704; **^{1}H NMR (DMSO-D$_6$, 250 MHz):** δ 12.63 (brs , NH_2) , 7.95 (s, 1H , H-8) , 7.38 (1 H, NH), 2.96 (s, 3H), 2.4 (s, 3H), **HRMS (ESI): m/z (M+H)** $^{+}$ calculated for $C_7H_{10}N_5$: 164.09307, found : 164.09296.

2-Methyladenine 12 (CAS: 1445-08-5)**: TX7**

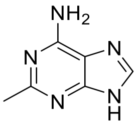

To a solution of compound **6** (100 mg, 0.593 mmol, 1 eq) in a 5:1 mixture of EtOH/H_2O (6.7 mL/ 1.35 mL) NaN_3 (115 mg, 1.77 mmol, 3 eq) was added. The mixture was stirred for2 h at 80°C and evaporated under reduced pressure. The crude product was dissolved in EtOH (8 mL) and $SnCl_2$, $2H_2O$ (400 mg , 1.77 mmol, 3 eq) was added. The solution was refluxed for 2 h and concentrated *in vacuo*. The product was purified by silica gel column chromatography (EtOAc / MeOH, 8/2).

White solid (74 %); **IR:** 3325, 3151, 2755, 2695, 1664 (NH_2), 1593, 1426, 1393, 1264, 930, 810, **^{1}H NMR (DMSO-D$_6$, 250 MHz):** δ 12.69 brs (NH), 7.96 (H-8), 6.94 (NH_2), 2.36 (CH_3); **HRMS (ESI): m/z (M+H)$^+$** calculated for $C_6H_8N_5$: 150.07742 found : 150.07725

1,7-dihydro-2-(trifluoromethyl)-6*H*-Purin-6-one 13 (CAS: 2268-14-6)**: TX8**

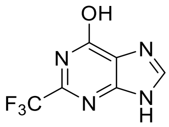

Trifluoroacetamide (174 g, 1.54 mol, 10 eq) was heated in an oil bath at 110°C. After the trifluoroacetamide melted, 50 g of 5-aminoimidazole-4-carboxamide hydrochloride (25.0 g, 0.154 mol) was added. The mixture was warmed to reflux (bath temp 160°C to 165°C) for 4 h, cooled to ambient temperature and the rocky solid was triturated with 1 L of ether and stirred for 2 h. The solid was collected by suction filtration. The solid was dispersed in water, stirred for 1 h and collected by suction filtration. The solid was dried under a vacuum.

Blue solid (48%),**IR:** 3151, 3034, 2939, 2800, 1727, 1687, 1376, 1336, 1190, 1143 (CF_3), 1108, 959, 786, **^{1}H NMR (DMSO-D$_6$, 250 MHz): δ** 13.75 (br s, 1 H, NH), 8.40 (s, 1 H), **^{19}F NMR (DMSO-D$_6$, 235 MHz):** δ -67.76 (s, 3F), **HRMS (ESI): m/z (M+H)$^+$** calculated for $C_6H_4N_4OF_3$: 205.03317 found : 205.3346

6-Chloro-2-trifluoromethyl-9*H*-purine 14 (CAS: 1998-63-6)**: TX9**

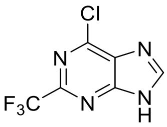

A mixture of 1,9-dihydro-2-(trifluoromethyl)-6H-purin-6-one (5.00 g, 24.5 mmol) and chloroform (100 mL) was refluxed with stirring. A solution prepared by dropwise addition of thionyl chloride (8.9 mL,

122 mmol, 5 eq) to cold dimethylformamide (8.92 g, 122 mmol, 5 eq) was added to the refluxing mixture. After 1.5 h at reflux, the reaction was poured into ice water (400 mL). The layers were separated, and the chloroform phase was washed with water (4x 100 mL). The pH of the aqueous phase was adjusted to 7 with saturated sodium bicarbonate and extracted with ether (3x 400 mL). The combined ether and chloroform extracts were dried with $MgSO_4$ and concentrated to dryness under reduced pressure .

White solid (64%), **IR:** 3 119, 3069, 2961, 2929, 2806, 1358, 1196, 1142 (CF_3), 1000, 872 ; **^{1}H NMR (DMSO-D$_6$, 250 MHz):** δ 14.45 (brs, 1 H, NH), 8.95 (s, 1 H, H-8); **^{19}F NMR (DMSO-D$_6$, 235 MHz)** :δ - 67.30 (s, 3F); **HRMS (ESI): m/z (M+H)** $^+$ calculated for $C_6H_3N_4F_3Cl$: 222.99928, found : 222.99912.

6-methoxy-2-(trifluoromethyl)-1H-Purine <u>15</u> (CAS: 658707-77-8)**: TX10**

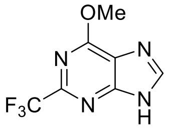

A 0.5-2 mL vial was loaded a solution of 6-chloro-2-trifluoromethyl-9H-purine (0.150 g, 0.673 mmol, 1 eq) and NaOH (81 mg, 2 mmol, and 3 eq) in methanol (1, 37 ml). After microwaving at 100°C for 40 min, the volatiles were distilled under reduced pressure; the resulting residue was then purified by silica gel chromatography. (EtOAc/ MeOH).

White solid (79 %), **IR:** 3119, 3005, 2951, 2781, 1628, 1391, 1285, 1138, 1117, 921, 804; ^{1}H NMR (DMSO-D$_6$, 250 MHz): 8.62 (s, 1H), 4.15 (s, 3H) ; **^{19}FNMR (DMSO-d$_{6, 235}$)** : -67.68; **HRMS (ESI): m/z (M+H)** $^+$ calculated for $C_7H_6N_4F_3O$: 219.04882 found : 219.04871.

***N*-methyl-2-(trifluoromethyl)-1*H*-Purin-6-amine <u>16</u>** (CAS: 18925-07-0): **TX11**

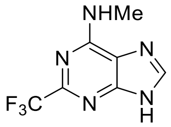

A 2-5 mL vial was charged with 6-chloro-2-trifluoromethyl-9H-purine (0.100 g, 0.45 mmol, 1 eq), *n*-butanol (0.90 mL), methylamine (0.106 mL, 2.66 mL, 6 eq), and distilled triethylamine (0.188 mL, 1.33 mmol, 3 eq). After microwave at 140 °C for 15 min, the solvent was removed under pressure, and the residue was washed with EtOAc. The product was dried under reduced pressure.

White solid (53%), **IR:** 2974, 2942, 2601, 1628, 1474, 1444, 1396, 1226, 1142, 1035, 936; **^{1}H NMR (DMSO-D$_6$, 250 MHz):** *δ* 8.25 (s, 1H), 8.15 (s, 1H), 3.05 (s, 3H); **^{19}F NMR (DMSO-D$_6$, 235 MHz)**: δ -68.06 (s); **HRMS (ESI): m/z (M+H)$^+$** calculated for $C_7H_6N_5F_3$: 218.06480 found : 218.06477.

2-(Trifluoromethyl)adenine <u>17</u> (CAS: 2993-06-8)**: TX12**

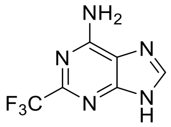

To a solution of 6-chloro-2-trifluoromethyl-9H-purine (0.150 g, 0.673 mmol, 1eq) in a 5:1 mixture of EtOH/H_2O (7.5 mL / 1.5 mL), NaN_3 (131 mg, 2 mmol, 3 eq) was added. The mixture was stirred for 2 h at 80°C and evaporated under reduced pressure. The crude product was dissolved in EtOH (9 mL) and $SnCl_2$, 2H20 (456 mg, 2 mmol, 3 eq) was added. The solution was refluxed for 2 h and concentrated in vacuo. The product was washed with EtOAc and dried under reduced pressure.

White solid (67%), **IR:** 3345, 3154, 3068, 3033, 2850, 1668, 1615, 1436, 1189, 1141, 1129, 1052, 950, 805; **^{1}H NMR (DMSO-D$_6$, 250 MHz):** δ 13.52 (brs, 1H, NH), 8.34 (s, 1H, H-8), 7.79 (s, 2 H, NH_2); **^{19}F NMR (DMSO-D$_6$, 235 MHz):** δ -67.9 (s, 3F) ;**HRMS (ESI): m/z (M+H)$^+$** calculated for $C_6H_5F_3N_5$: 204.04915 found : 204.04890.

2-(trifluoromethyl)-Purine-6-thiol <u>18</u> (CAS: 1996-33-4)**: TX13**

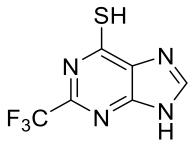

A solution of **<u>14</u>** (200 mg, 1.19 mmol, 1 eq) and thiourea (397 mg, 5.24 mmol, 4.42 eq) in ethanol was stirred at reflux for 4 h. After evaporation, the residue was purified by silica gel column chromatography (EtOAc/MeOH, 8:2).

Beige solid (54%), **IR:** 3348, 2724, 2358, 2196, 1652, 1580, 1551, 1418, 1356, 1191, 1137, 1007, 892, **^{1}H NMR (MeOH-D$_6$, 250 MHz)**: δ 8.25 (s, 1H). **HRMS (ESI): m/z (M+H)$^+$** calculated for $C_6H_4F_3N_4S$: 221.01032, found : 221.01035.

2-THIOXANTINE <u>19</u> (CAS: 2487-40-3) (Commercial) **: 2TX**

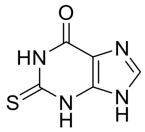

The compound 4,5-diamino-6-hydroxy-2-mercaptopyrimidine (MW 158, Aldrich 95%, used as supplied, 500 mg) was refluxed with 90% formic acid (50 mL) for 2 h in a 3-neck flask with mechanical stirring. The mixture initially solidified and an additional 0.5 mL formic acid was added. The mixture was cooled on ice and crude 4-amino-5-formamido-6-hydroxy-2-mercaptopyrirnidine was filtered on a large sinter. It was covered with a rubber dam and dried under vacuum for 30 min giving a light-yellow product. The filtration cake was suspended in formamide (1.12 mL) in a large beaker and heated at 175-185 °C (bath temperature) in a liquid paraffin bath with occasional hand stirring for 2 h. Considerable frothing occurred in the early stages. The mixture was cooled to room temperature and filtered with a dam on a large sinter, then dissolved in approximately 10 mL of 1M NaOH and precipitated with glacial AcOH at 10 °C. It was then filtered, pressed dry and vacuum dried at 95 °C for 2 h.

Light yellow Solid (97%), **IR:** 3585, 3100, 1678, 1565, 1422, 1209, 977, 950, 804, 517, **^{1}H NMR (DMSO-*d*$_6$, 250 MHz):** δ 13.40 (s, 2H), 12.19 (s, 1H), 8.06 (s, 1H); **HRMS (ESI): m/z (M+H)$^+$** calculated for $C_5H_5N_4OS$: 169.017858, found :169,01162.

2-Mercapto-6, 8-purinediol <u>20</u> (CAS: 15986-31-9)**: TX14**

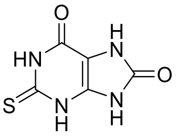

The compound 5,6-diamino-2-thiouracil (1g, 6.3 mmol, 1 eq) and urea (1.96 g, 32.7 mmol, 5 eq) were mixed in a flask with stirring and heated to 170 °C for 30 min. The agitation became difficult. Sodium hydroxide was added to dissolve all solid material. The hot solution was filtered. The hot filtrate was acidified with concentrated hydrochloric acid. The precipitate was filtered and washed by water and acetone.

Yellow Solid (90%), **IR:** 3675, 2987, 1933, 1589, 1505, 1465, 1250, 1066, 858, 656, 544**.** , **^{1}H NMR (DMSO-D$_6$, 250 MHz):** δ 12.19 (s, 1H), 11.51 (s, 1H), 11.00 – 10.76 (s, 1H). **HRMS (ESI): m/z (M+H)$^+$** calculated for $C_5H_5N_4O_2S$:185.01277, found : 185.01300.

6, 7 –dimethyl-4-hydroxy-2-mercaptopterdine <u>21</u> (CAS: 54030-51-2)**: TX15**

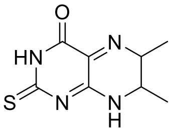

The compound 5,6-diamino-2-thiouracil (0.58 g, 3.67 mmol) was dissolved in DMF (20 mL) and diacetyl (0.63 g, 7.34 mmol) was added. The solution was stirred for 5 h at 100 °C, and then the solvent was evaporated under reduced pressure. The resulting orange oil was triturated with ether. The resulting solid was filtered and washed with ether.

Beige Solid (67%), **<u>IR</u>**: 3675, 3188, 2987, 1694, 1539, 1393, 1378, 1270, 1127, 1065, 502 ; **^{1}H NMR (DMSO-d$_6$, 250 MHz) δ** 13.13 (s, 1H), 12.60 (s, 1H), 2.56 (s, 3h), 2.53 (s, 3h) ;**HRMS (ESI): m/z (M+H)$^+$** calculated for $C_8H_9N_4OS$: 209.04915, found :209.04936.

1,2,3,6,7,9-hexahydro-2,6-dithioxo-8H-Purin-8-one <u>22</u>(CAS : 6703-93-1)**: TX16**

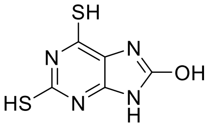

The compound 4,5-diamino-2,6-dimercaptopyrimidine (1.72 mmol, 1eq) and urea (8.95 mmol, 5.2 eq) were mixed in a flask with stirring and heated to 170 ° C for 45 min. The agitation became difficult. Potassium hydroxide was added to dissolve all solid material. The hot solution was treated with Norit and filtered. The hot filtrate was acidified with concentrated hydrochloric acid. The precipitate was filtered and washed by water.
Yellow solid (60%), **IR:** 3006, 1694, 1584, 1486, 1358, 1302, 1169, 1132, 990, 840, 721; **HRMS (ESI): m/z (M+H) + calcd for** $C_5H_5N_4OS_2$: 200.98992, found: 200.99019.

3-Methyl-8-phenyl-2-thiohypoxanthine 23 (CAS: 103258-00-0)**: TX17**

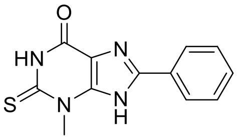

A mixture of 5, 6-diamino-1-methyl-2-thioxo-2,3-dihydro-1H-pyrimidin-4-one (1 eq) and (triethoxymethyl)benzene (10 eq) was heated under reflux for 15h. Cooling the solution yielded the desired product.

Yellow Solid (90 %), **IR**: 3675, 3126, 2987, 1678, 1405, 1217, 1171, 1066, 781, 703 ; **^{1}H NMR (DMSO-d$_6$, 250 MHz)** δ 3.74 (s, 3H), 2.36 (s, 3H) ; **HRMS (ESI): m/z (M+H)$^+$** calculated for $C_{12}H_{11}N_4OS$: 259.06481, found: 259.06479.

3, 8-dimethyl-2-thioxanthine 24 (CAS : 91725-06-3)**: TX18**

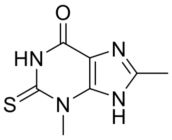

A mixture of 5, 6-diamino-1-methyl-2-thioxo-2, 3-dihydro-1H-pyrimidin-4-one (1 eq) and an triethylorthoacetate (10 eq) was heated under reflux for 15h. Cooling the solution yielded the desired product.

Yellow solid (60%), **IR**: 3675, 2987, 1405, 1250, 1065, 891, 510, 501 ; **^{1}H NMR (DMSO-d$_6$, 250 MHz)** δ 3.74 (s, 3H), 2.36 (s, 3H) ; **HRMS (ESI): m/z (M+H)$^+$** calculated for $C_7H_8N_4OS$: 197.049158, found :197.049281.

● Supplementary tables

Table S1: Amino acid residues of free and DNA bound LlFpg and hNeil1 involved in the docking site areas for 2TX$_{Red}$ and TX19$_{Red}$

	Site I	Site II
**Fpg/DNA**	P1 E2 P4 E5 V6 T8 T83 D85 A86 P87 R88 E89 V108 I172 G215 G216 S217 S218 I221 Y222 S223 A224 L225 GG226 S227 T228	K154 P158 Y159 L161 E162 Q163 A258
**Free Fpg**	P1 E2 P4 E5 V6 V9 G216 S217 S218 I219 A224 L225 G226 S227	R55 G56 K57 Y58 K129 I131 Q163 G168
**hNEIL1/DNA**	E5 Y176 V235 V236 Q237 L238 G239 G240 R241 Y243 G244 S245 G248 E249 D251 F252 A253 F255	R158 C161 E162 A163 L165 D166 Q167 R168 G174 D272 R273 H274 R276
**Free hNEIL1**	L6 G239 Y243 G244 S245 E246 F252 A253 F255 R256	K53 E54 L165 D166 Q167 F170 N171 G172 I173 G174 N175

Table S2: Amino acid residues of free and DNA bound Fpg and hNeil1 defined the area of docking sites I, II and III for the disulfide forms 2TX$_{Ox}$ and TX19$_{Ox}$ (TX19$_{Ox1}$ and TX19$_{Ox2}$, see Figure 6)

	Site I	Site III	Site II
**Fpg/DNA**	P1 E2 S217 S218 I219 R220 T221 Y222 S223 A224	-	Y159 L160 L161 E162 Q163 T164 L165 V166 A167 G168 L169 G170 V257 A258 G259 R260
**Free Fpg**	**P1 E2** M75 E76 G216 S217 S218 I219 G226 Q232	**P1 E2 K57** M75 G170 N171 I172	**K57** K129 K130 I131 G132 P133 G134 P135 E162 Q163 A167 G168 L169
**hNEIL1/DNA**	P1 E2 E5 V235 V236 Q237 L238 G239 G240 R241 G242 Y243 G244 S245 G248 E249 F252 A253 F255	-	L165 D166 Q167 R168 F169 F170 N171
**Free hNEIL1**	**P1 E2** L8 S10 S83 F84 G239 G240 G244 S245 E246 G248 E249 A253 F252 F255	**P1 E2** E3 E5 L6 **K53** **N175** Y176 L177 P232 K233 V236 Q237	**K53** E54 L55 D166 Q167 R168 G172 I173 G174 **N175**

The site III is observed only with TX19$_{Ox}$ and is not accessible in the enzymes bound to DNA. Amino acids in bold are shared by the site III and the other binding sites.

Table S3: X-ray data collection and refinement statistics

	2TX	TX13	TX15	TX19	TX19 (TCEP)	TX20	TX27
**Data collection statistics**							
Radiation source	PROXIMA-1	ID30A-3	PROXIMA-1	PROXIMA-1	PROXIMA-2	PROXIMA-1	ID30A-3
Wavelength (Å)	0.97857	0.96770	0.97857	0.97857	0.98010	0.97857	0.96770
$P4_12_12$ spacegroup with cell dimensions: $a = b$, c (Å)	91.36, 141.03	91.81, 141.62	91.67, 141.04	92.18, 139.73	91.63, 141.86	91.42, 140.28	91.96, 142.18
Resolution range (Å)	47.63-1.82 (1.92-1.82)	47.21-2.00 (2.04-2.00)	47.72-2.00 (2.11-2.00)	47.66-2.25 (2.37-2.25)	47.84-1.95 (2.00-1.95)	47.53-2.25 (2.37-2.25)	59.13-1.76 (1.79-1.76)
Total observations	352566 (50454)	292071 (15391)	239478 (34643)	106520 (15359)	213 417 (14586)	135979 (19588)	440572 (22689)
Unique reflections	53367 (7546)	41111 (2048)	41266 (5925)	29044 (4146)	44101 (3023)	28957 (4144)	61222 (3017)
Completeness (%)	98.8 (97.7)	99.1 (99.9)	99.8 (99.9)	99.4 (99.2)	99.0 (97.3)	99.9 (99.9)	99.9 (99.3)
Multiplicity	6.6 (6.7)	7.1 (7.5)	5.8 (5.8)	3.7 (3.7)	4.8 (4.8)	4.7 (4.7)	7.2 (7.5)
R_{merge}[a] (%)	5.2 (88.3)	5.6 (95.0)	6.4 (78.5)	6.0 (70.6)	6.4 (88.0)	4.8 (68.1)	5.6 (95.7)
Average $I/\sigma(I)$	18.4 (2.1)	18.7 (2.1)	13.5 (2.1)	14.3 (1.9)	14.7 (1.5)	17.3 (2.1)	18.3 (2.2)
$CC_{1/2}$ (%)	99.9 (73.0)	99.9 (66.6)	99.8 (72.9)	99.8 (99.2)	99.9 (64.8)	99.9 (73.3)	99.9 (75.6)
**Refinement and model statistics**							
Resolution range (Å)	47.63-1.82	45.91-2.00	45.83-2.00	41.57-2.25	47.84-1.95	47.53-2.25	59.13-1.76
Number of reflections used	53339	41035	41221	29004	44066	28910	61222
R_{work}[b] / R_{free}[c] (%)	15.58/17.23	17.39/19.50	16.58/18.55	16.35/20.19	16.75/19.56	16.00/19.79	16.92/18.75
Average B values (Å²)							
All atoms	43.3	50.1	52.6	53.3	37.4	62.1	38.5
Protein atoms	37.7	46.7	48.6	48.9	33.7	57.3	33.3
DNA atoms	60.0	63.5	67.0	72.7	47.6	81.6	50.2
Inhibitor atoms	74.1	59.9	51.0	39.3	38.6	57.0	30.0
Glycerol atoms	50.5	55.5	52.8	61.0	55.6	54.3	56.7
Water atoms	47.6	45.8	53.7	50.8	43.1	54.4	49.1
Root mean square deviation from ideality							
Bond lengths (Å)	0.007	0.008	0.008	0.008	0.016	0.011	0.010
Bond angles (°)	1.09	0.98	0.95	1.18	1.48	1.26	1.00
Ramachandran analysis (% of residues)							
Favoured regions / Allowed regions / Outliers	97.4/2.6/0.0	97.0/3.0/0.0	97.3/2.7/0.0	97.0/3.0/0.0	96.7/3.3/0.0	97.7/2.3/0.0	97.7/2.3/0.0
Number of atoms							
Protein	2117	2137	2105	2091	2168	2108	2211
DNA	557	557	557	696	557	557	557
Inhibitor	33	14	14	12	12	11	11
Glycerol atoms	6	6	6	6	12	6	12
Water atoms	281	112	177	126	312	75	449
**PDBid**	6RO2	6RNR	6RP7	6RP0	6ROK	6RNO	6RNM

[a] $R_{merge} = \sum_h \sum_i |I_{h,i} - \langle I \rangle_h| / \sum_h \sum_i I_{h,i}$ where $\langle I \rangle_h$ is the mean intensity of the symmetry-equivalent reflections.
[b] $R_{work} = \sum_h ||F_o| - |F_c|| / \sum_h |F_o|$, where F_o and F_c are the observed and calculated structure factor amplitudes, respectively, for reflection h.
[c] R_{free} is the R-value for a subset of 5% of the reflection data, which were not included in the crystallographic refinement.

• Supplementary figures (Figures S)

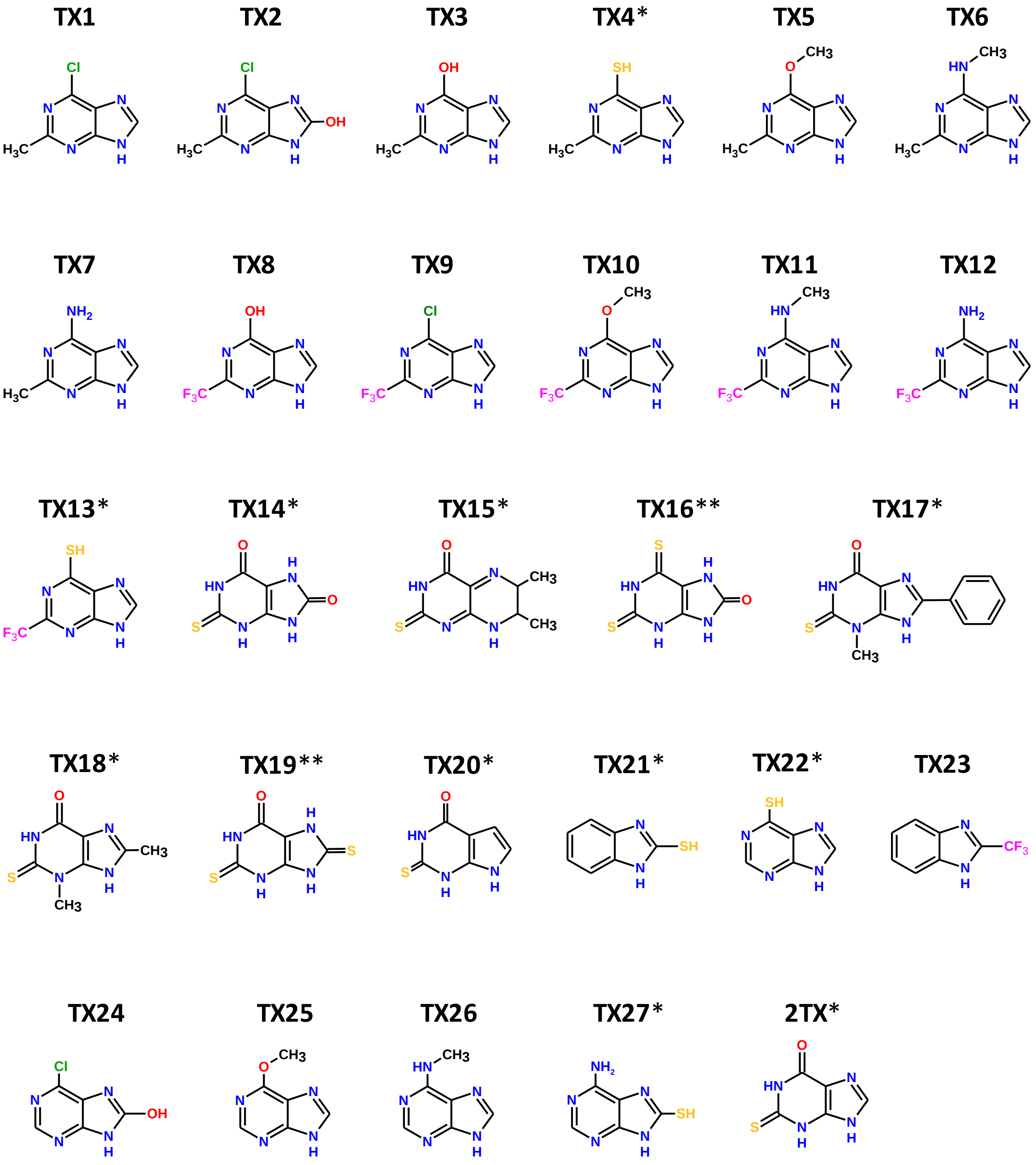

Figure S1. **Small library of thioxanthine derivatives** The design and synthesis are described in the supplementary information. * and ** indicate monothio- and dithio-compounds, respectively.

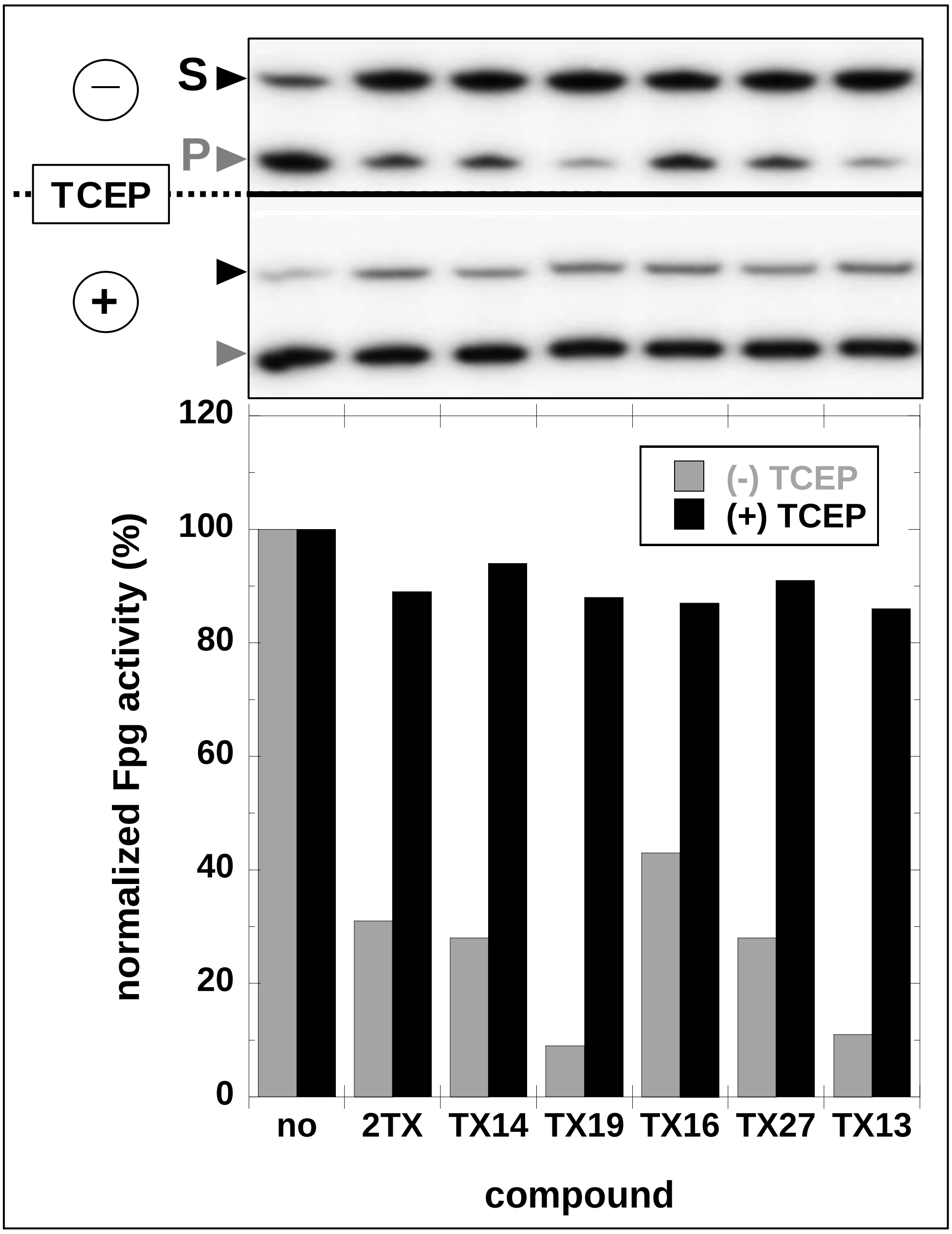

Figure S2. Effect of TCEP on 2TX and TXn-induced Fpg inhibition activity Fpg and 24-bp 8-oxoG-containing DNA duplex (S) were incubated as described in the Figure 1 caption, alone (lane 'no') or with 0.2 mM of 2TX and TXn inhibitors as indicated, in the presence (+) or absence (-) of 2 mM of TCEP. (P) indicates the Fpg DNA cleavage product. The assays were then analyzed by Urea-PAGE as described in the 'Materials and Methods'. Representative Urea-PAGE autoradiography and corresponding quantification are shown.

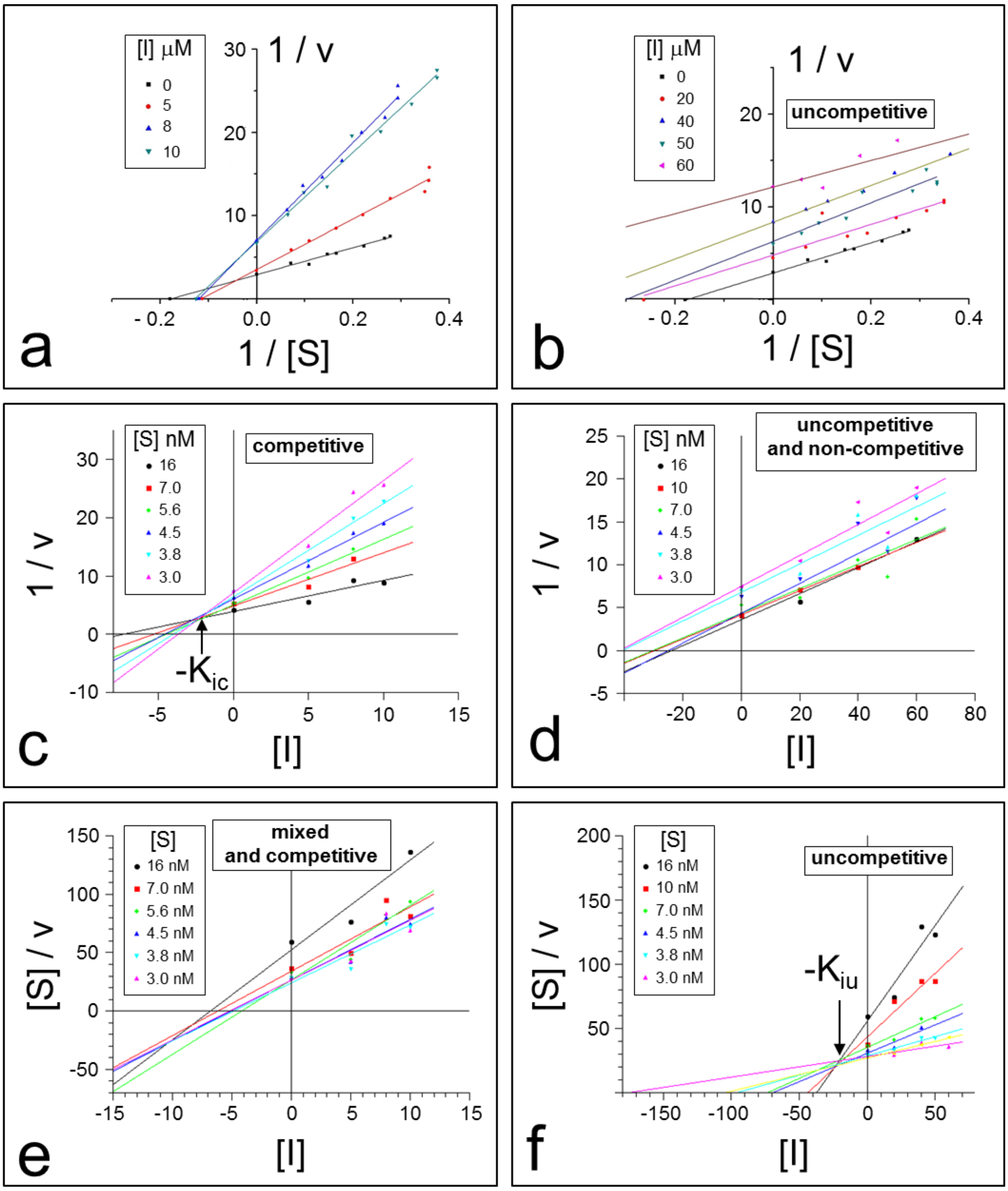

Figure S3. Double-reciprocal plots for the effects of 2TX on the velocity of *Ll*Fpg catalyzed the excision of 8-oxoG I (inhibitor) indicates 2TX and S represents the enzyme substrate, a 24-mer DNA duplex containing one 8-oxoG opposite cytosine (see the 'Materials and Methods'). K_{iu} and K_{ic} correspond to the inhibition constants. **(a)** and **(b)** Lineweaver-Burck plots, **(c)** and **(d)** Dixon plots and **(e)** and **(f)** Cornish-Bowden plots for low and high 2TX concentrations, respectively. The lines drawn are obtained by applying the following equations:

- for (a) and (b): $1/v = 1/V_{max} + (1/[S].K_m/V_{max}).(1+[I]/K_i)$;
- for (c) and (d): $1/v = [(1+K_m/[S])/(V_{max}.K_i)].[I] + 1/V_{max}.(1+K_m/[S])$;
- for (e) and (f): $[S]/v = K_m/V_{max}.(1+[I]/K_i) + [S]/V_{max}.(1+[I]/K_{iu})$

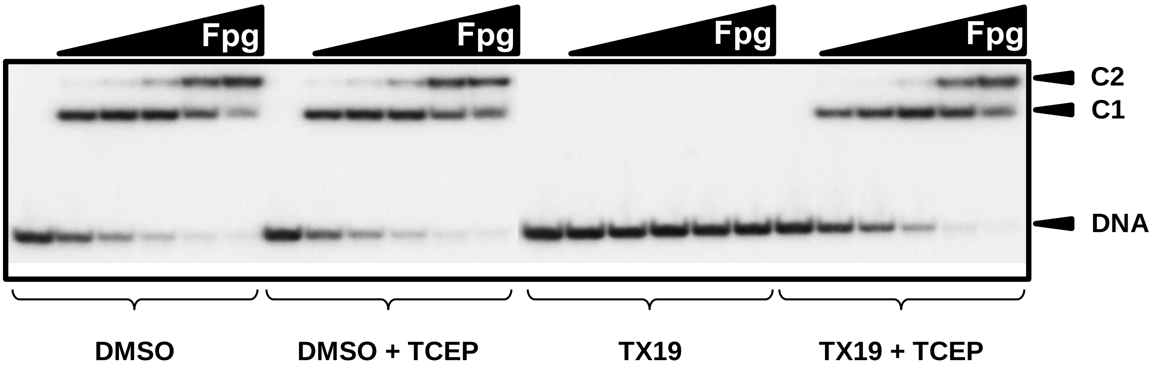

Figure S4. Effect of TCEP on the Fpg DNA binding properties in the presence of TX19
0.1 nM of 5'[^{32}P]-THF-containing 14-mer DNA duplex was incubated with 0, 0.25, 0.5, 1, 5 or 25 nM Fpg alone or after 30 min Fpg preincubation at 4°C with 0.3 mM TX19 and in the presence or absence of 1 mM TCEP. At equilibrium, incubation mixtures were analyzed by EMSA as described in the 'Materials and Methods'.

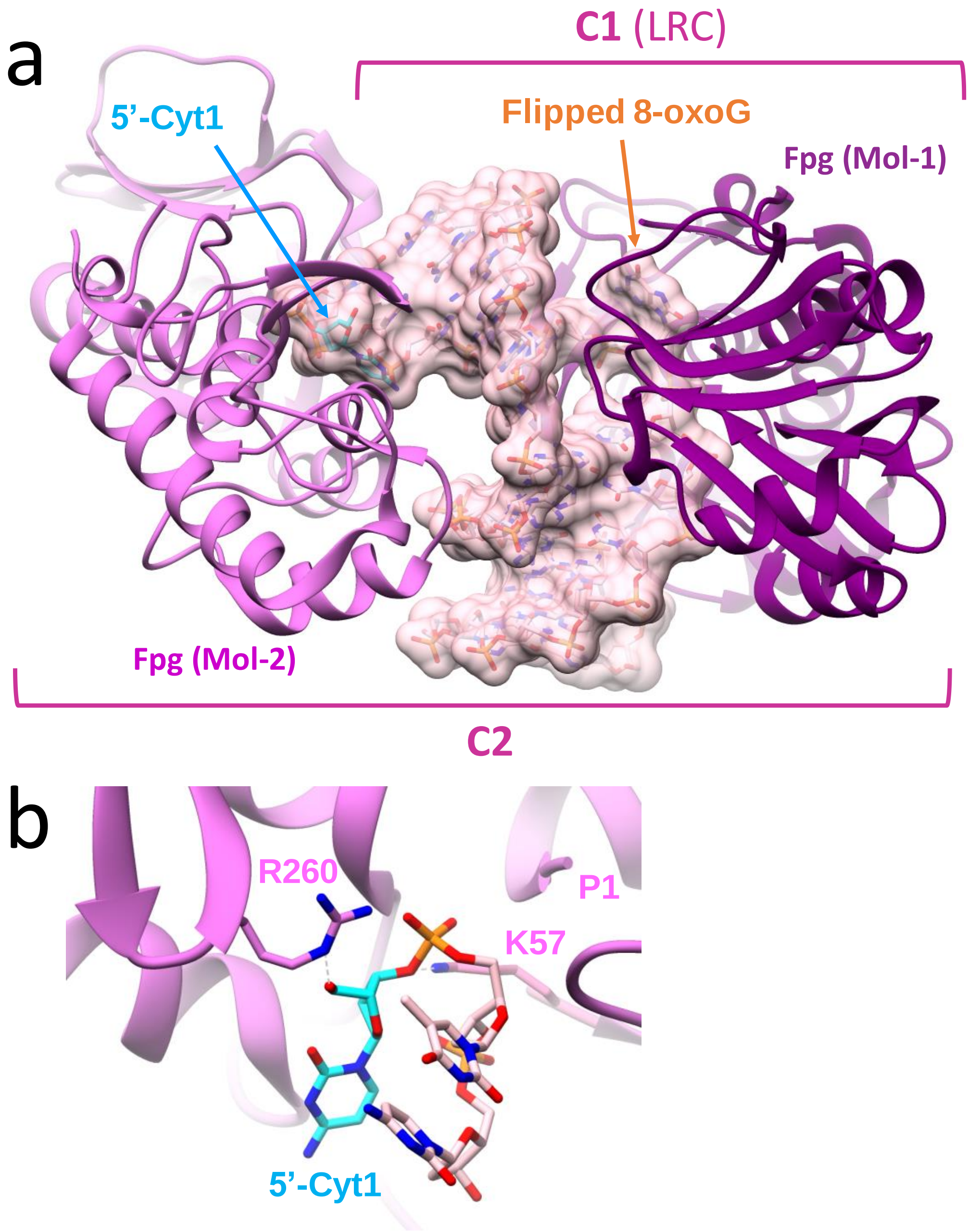

Figure S5. Crystal structure of LlFpg bound to 14-mer c8-oxoG:C DNA duplex (PDB id code 4CIS) **(a)** An overview. **(b)** Zoom of the interaction of a second Fpg molecule (Mol-2). Mol-2 binds to the overhanging 5'-cytosine-containing damaged strand (5'-Cyt(1)) following the binding of a first Fpg molecule (Mol-1) on the base damage 8-oxoG (C1, LRC, lesion recognition complex). C2 might correspond to the C2 complex observed in EMSA experiments with LlFpg (see text). c8-oxoG indicates the carbanucleoside analog of 8-oxoG.

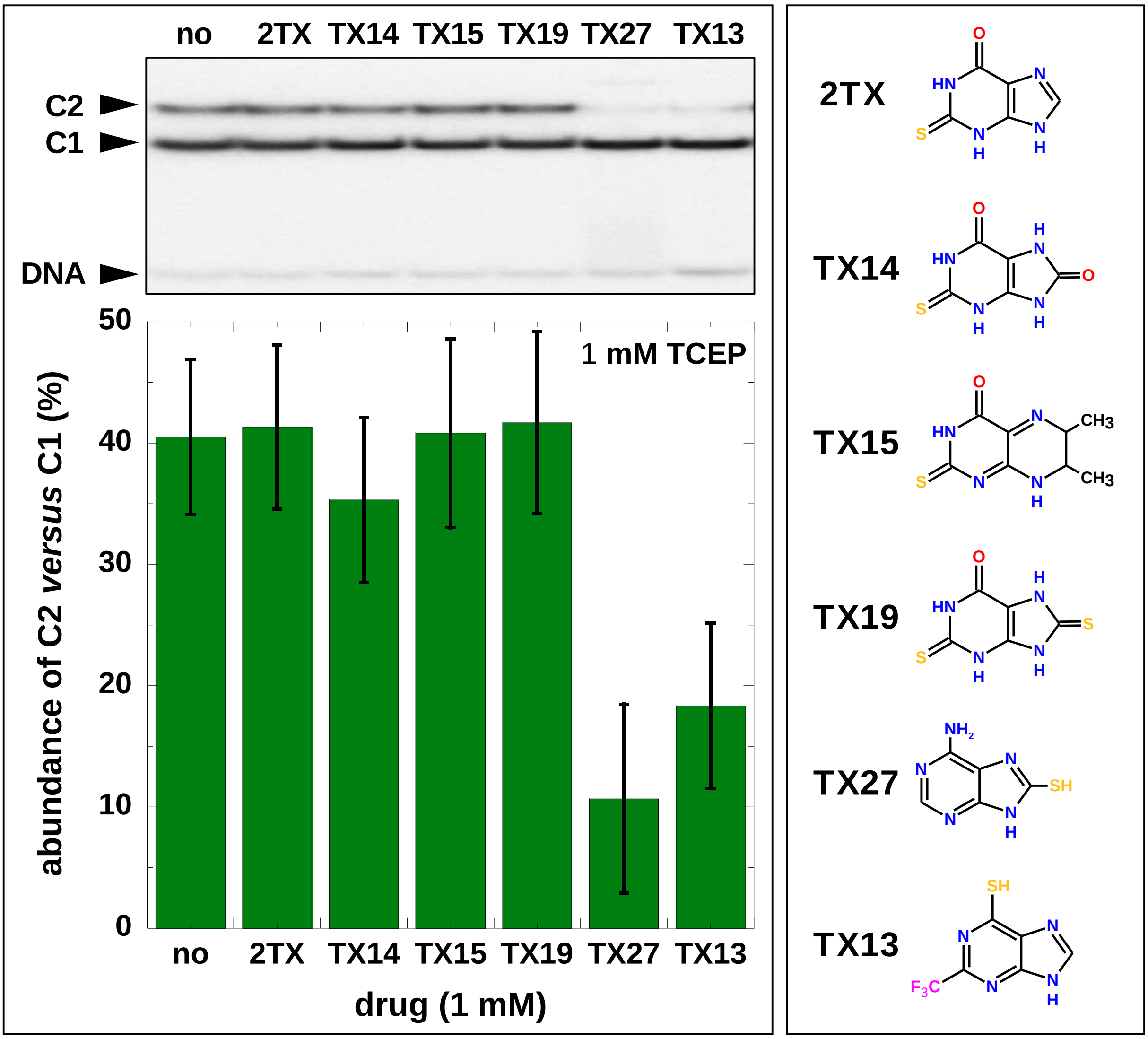

Figure S6. Destabilization of the C2 Fpg/DNA complex by TX13 and TX27 under moderate reducing conditions *Left panel*: at the top is a representative autoradiography of an EMSA experiment that shows the effect of the indicated thiocompounds (1 mM) on the C2 complex. Binding experiments were performed with a limited Fpg concentration, namely the amount required to form approximately 40% of C2; at the bottom: EMSA quantification from at least three independent experiments. *Right panel*: chemical structures of 2TX/TXn used in this experiment.

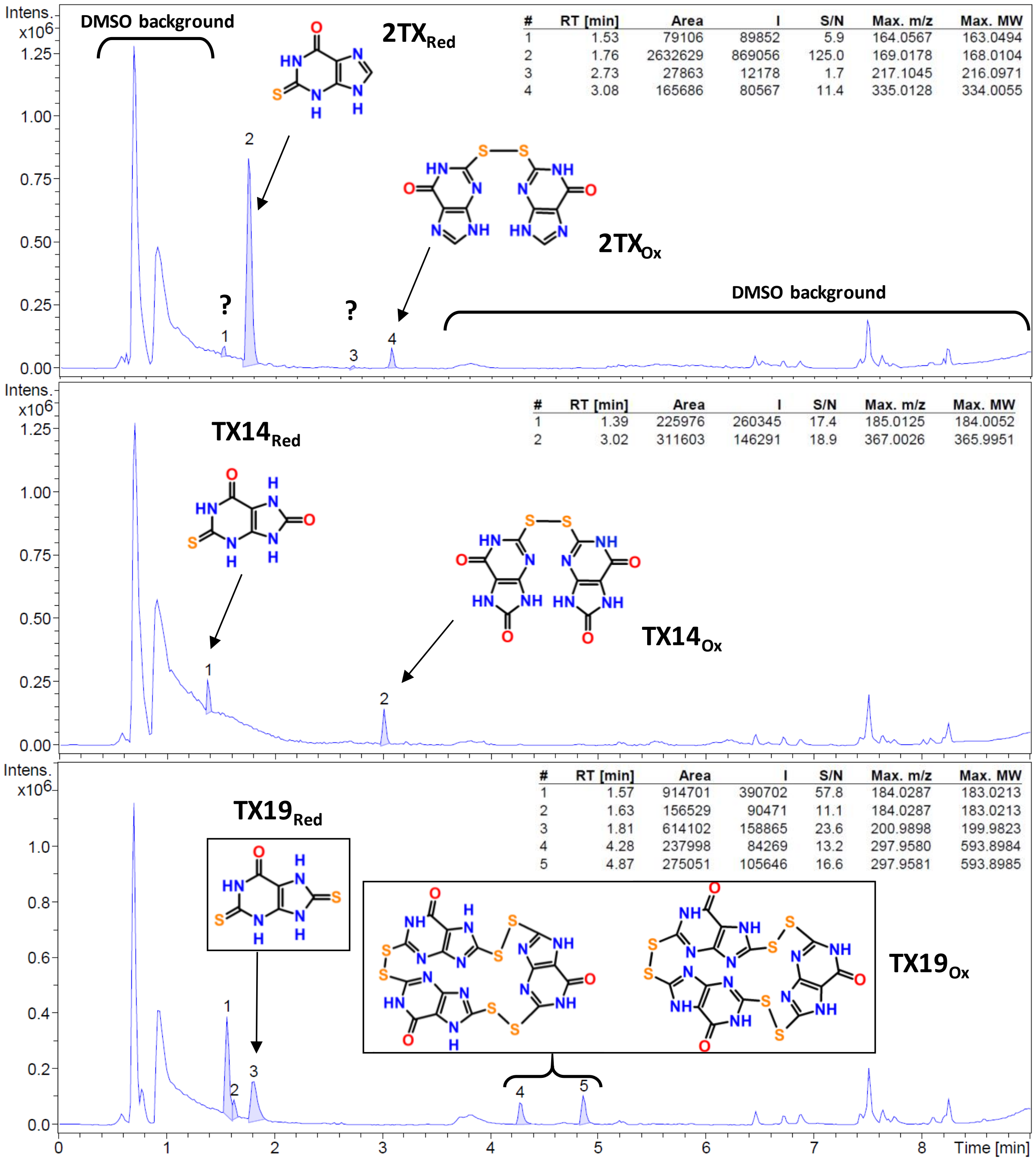

Figure S7. LC-HRMS spectra of 2TX, TX14 and TX19 Stock solutions in 80% DMSO were stored at -80°C. An aliquot of each sample at 1.2 mM was freshly thawed and diluted 1:28 with water and loaded onto a Zorbax Eclipse Plus C18 column (2.1x150 mm, 1.8 mm; Agilent). LC conditions are described in the *'Materials and Methods'*. The peaks highlighted by a *blue* background correspond to molecules present in stock solutions of 2TX, TX14 and TX19. The other peaks are related to a background noise of DMSO ions partly eluted in the dead volume of the column (proximal peaks) or resolved by the column (distal peaks).

a

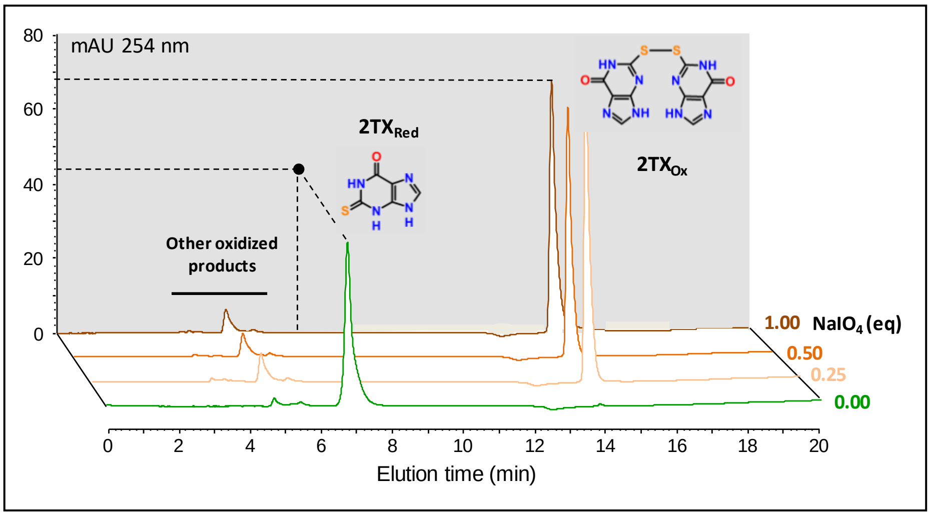

b

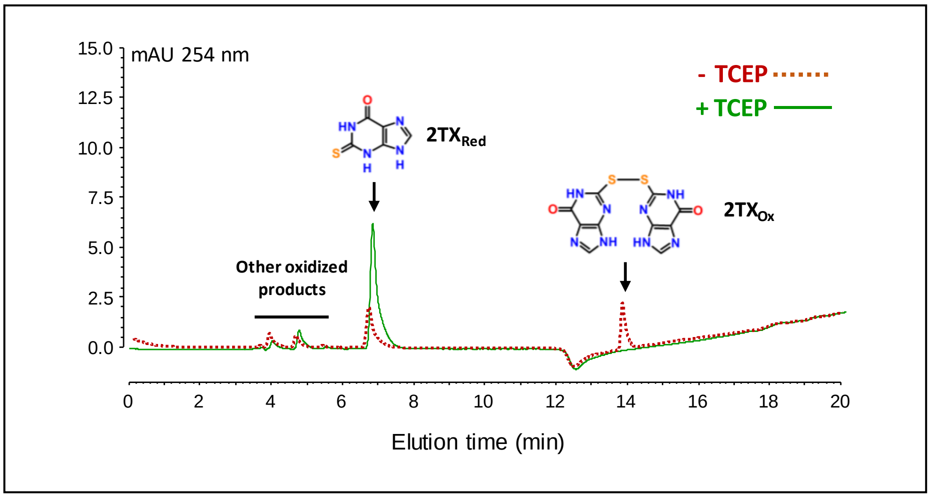

Figure S8. Preparation of $2TX_{Ox}$ and $TX14_{Ox}$ Reduced forms of 2TX (TX14) were incubated with $NaIO_4$ and the resulting incubation mixture were then analyzed by HPLC on an RP-C4 column. The isolated oxidized species were analyzed by LC-HRMS as described in the '*Materials & Methods'* . **(a)** Oxidation reaction of one equivalent of $2TX_{Red}$ followed by HPLC as function of $NaIO_4$ equivalent added. **(b)** Effect of TCEP on the chromatogram profile showing the interconversion of $2TX_{Ox}$ in $2TX_{Red}$.

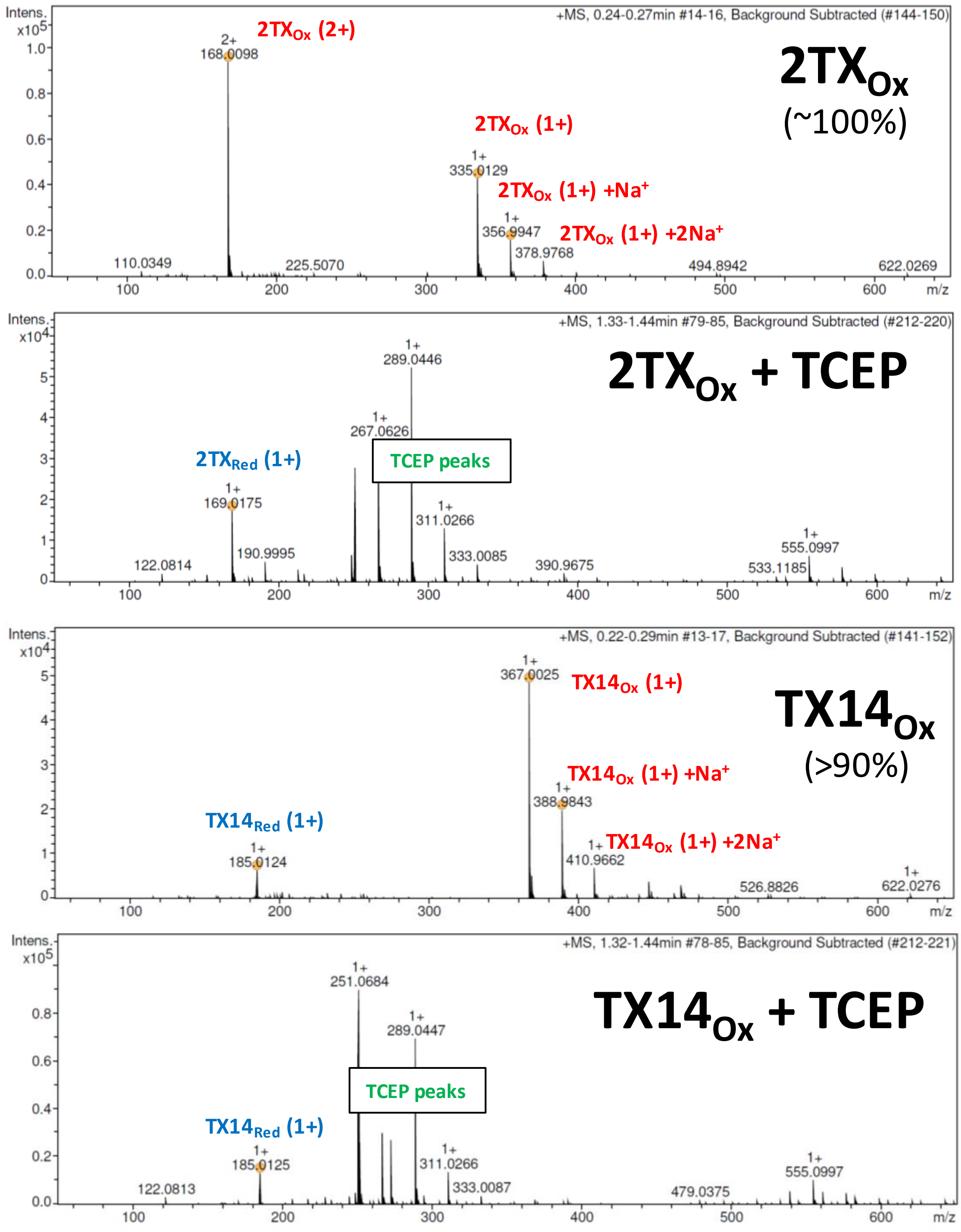

Figure S9. High-resolution mass spectra of purified $2TX_{Ox}$ and $TX14_{Ox}$ +/- TCEP

Figure S10. Blind and flexible docking of $TX19_{Ox}$ in pictures

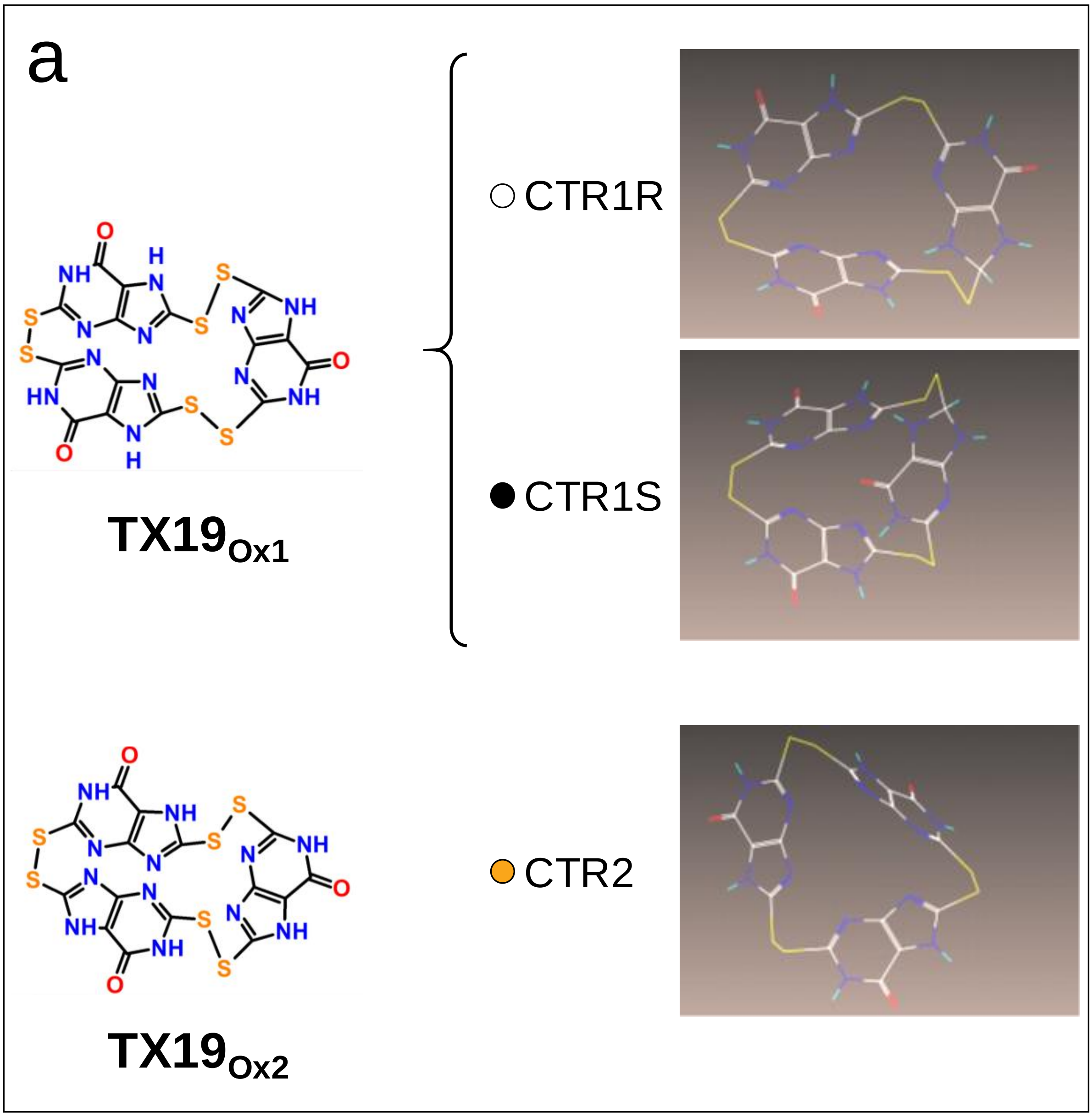

Figure S10a: ***Structures of $TX19_{Ox}$*** There are two possible combinations for the disulfide trimeric cyclic forms of TX19 ($TX19_{Ox1}$ and $TX19_{Ox2}$) that correspond to the two species identified by LC-HRMS (**Figure S7**). The presence of an asymmetric centre in $TX19_{Ox1}$ generates the possibility of two possible configurations: CTR1 'R' and 'S'. For $TX19_{Ox2}$, there is only one possible configuration, namely CTR2. Several representative conformers (considering the intrinsic dynamic and major tautomers at physiological pH) of each TX19 disulfide form were generated and used for blind docking on each conformation extracted from MD simulations (centroids Cn, **Figure 7**) of free and DNA bound LlFpg and hNeil1.

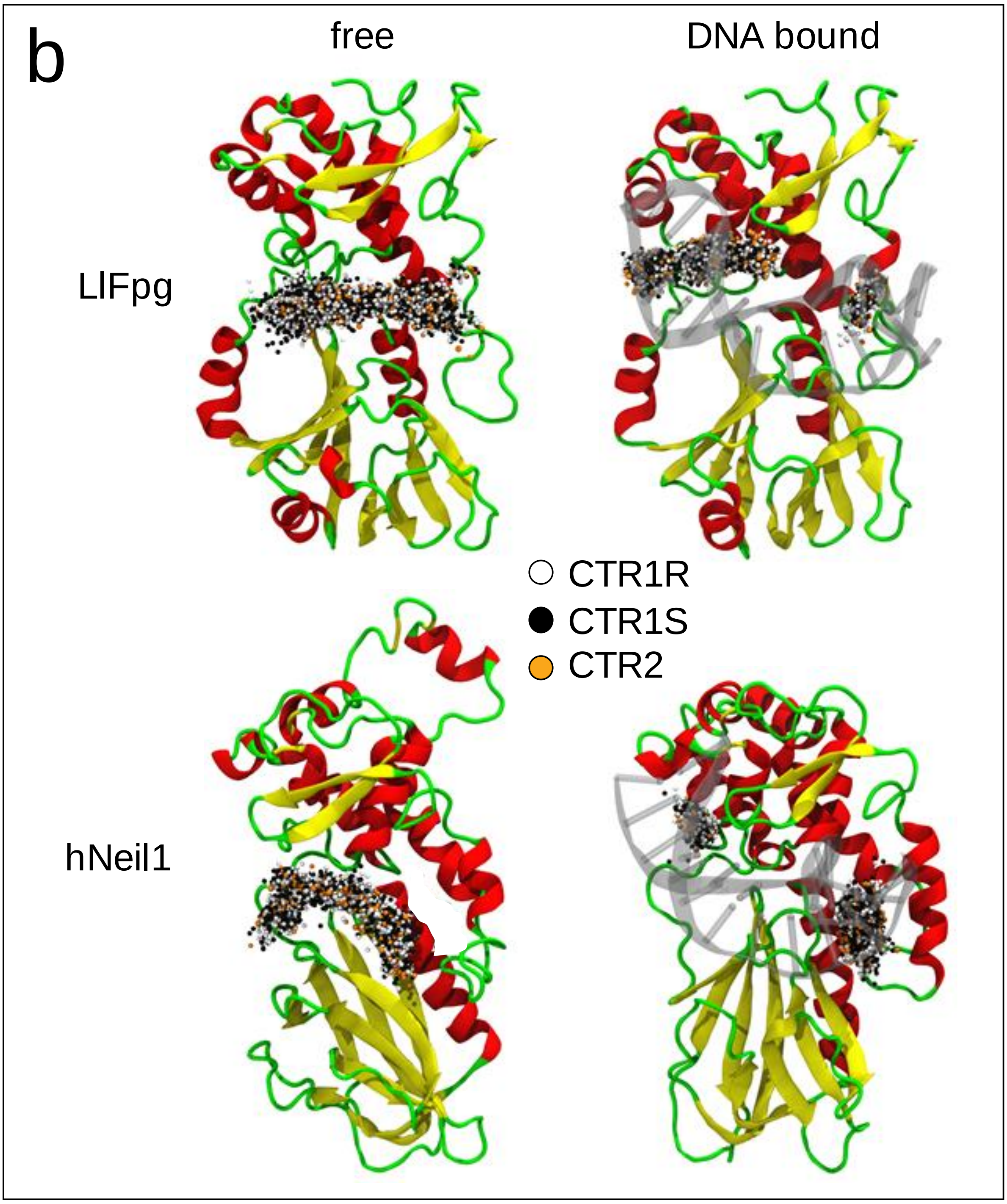

Figure S10b: ***Overview of the docking of disulfide forms of TX19 on free and DNA bound enzymes*** Ribbon presentations correspond to the crystal structures of the free and DNA bound Fpg and hNeil1 as indicated. Alpha-helices and beta-strands are coloured in *red* and *yellow*, respectively. Each docking poses obtained with the conformers of the cyclic trimeric disulfide forms of TX19 (CTR1R, CTR1S and CTR2, as indicated) are shown by small spheres that correspond to the geometric centre of the considered docked ligand.

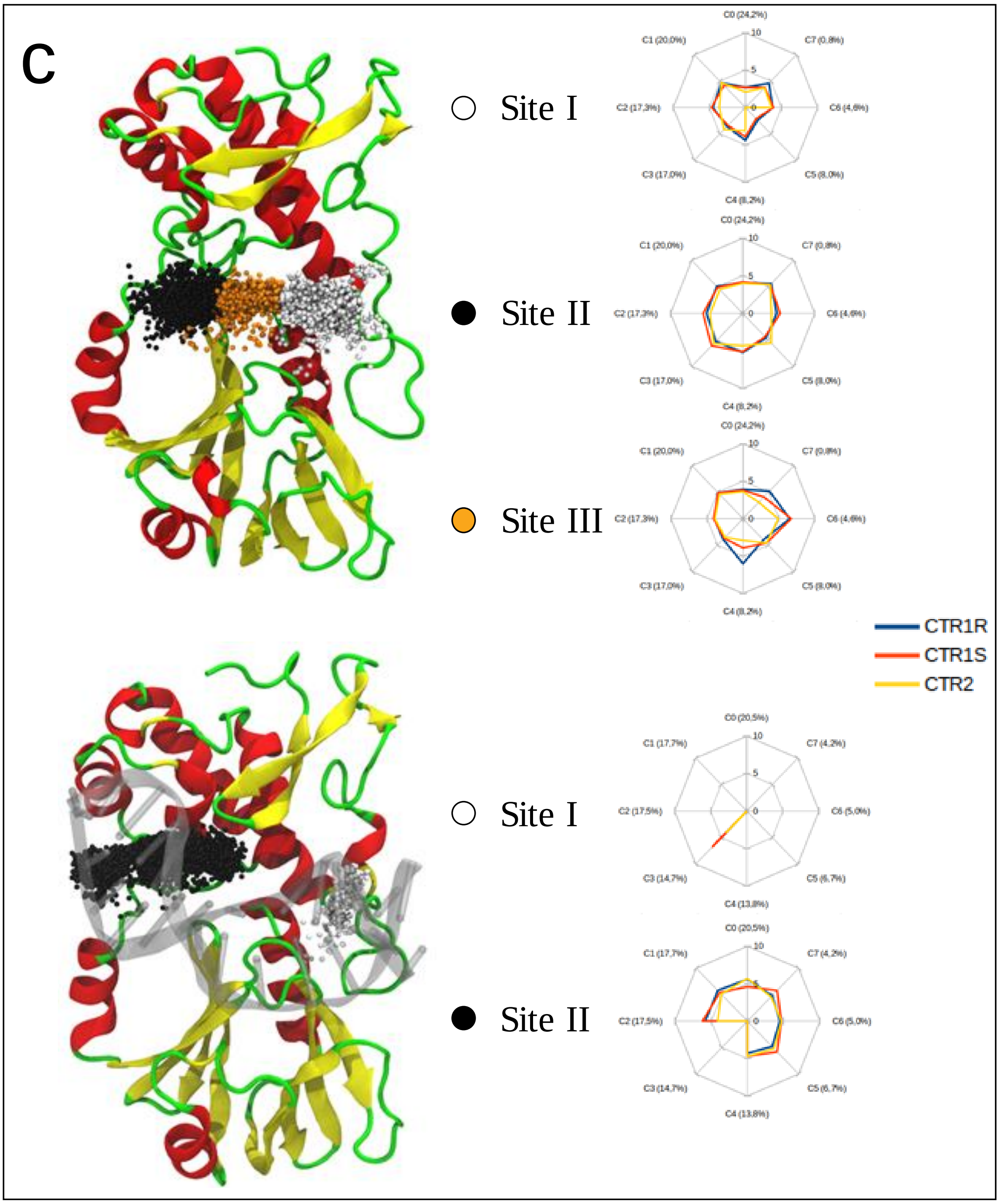

Figure S10c: ***Docking of disulfide forms of TX19 on free and DNA bound Fpg visualized on the preferential docking sites I, II and III*** Ribbon presentations correspond to the crystal structure of free and DNA bound Fpg (1PM5 without DNA and 1PM5, respectively). DNA is colored in *grey*. Docking poses obtained with the conformers of the cyclic trimeric disulfide forms of TX19 (CTR1R, CTR1S and CTR2, as indicated) are shown by small spheres that correspond to the geometric centre of the ligand. The docking pose scores obtained for sites I, II and III are plotted as function of the centroids Cn extracted from MD simulations using Kiryat (Radar) charts presented on the right of the figure.

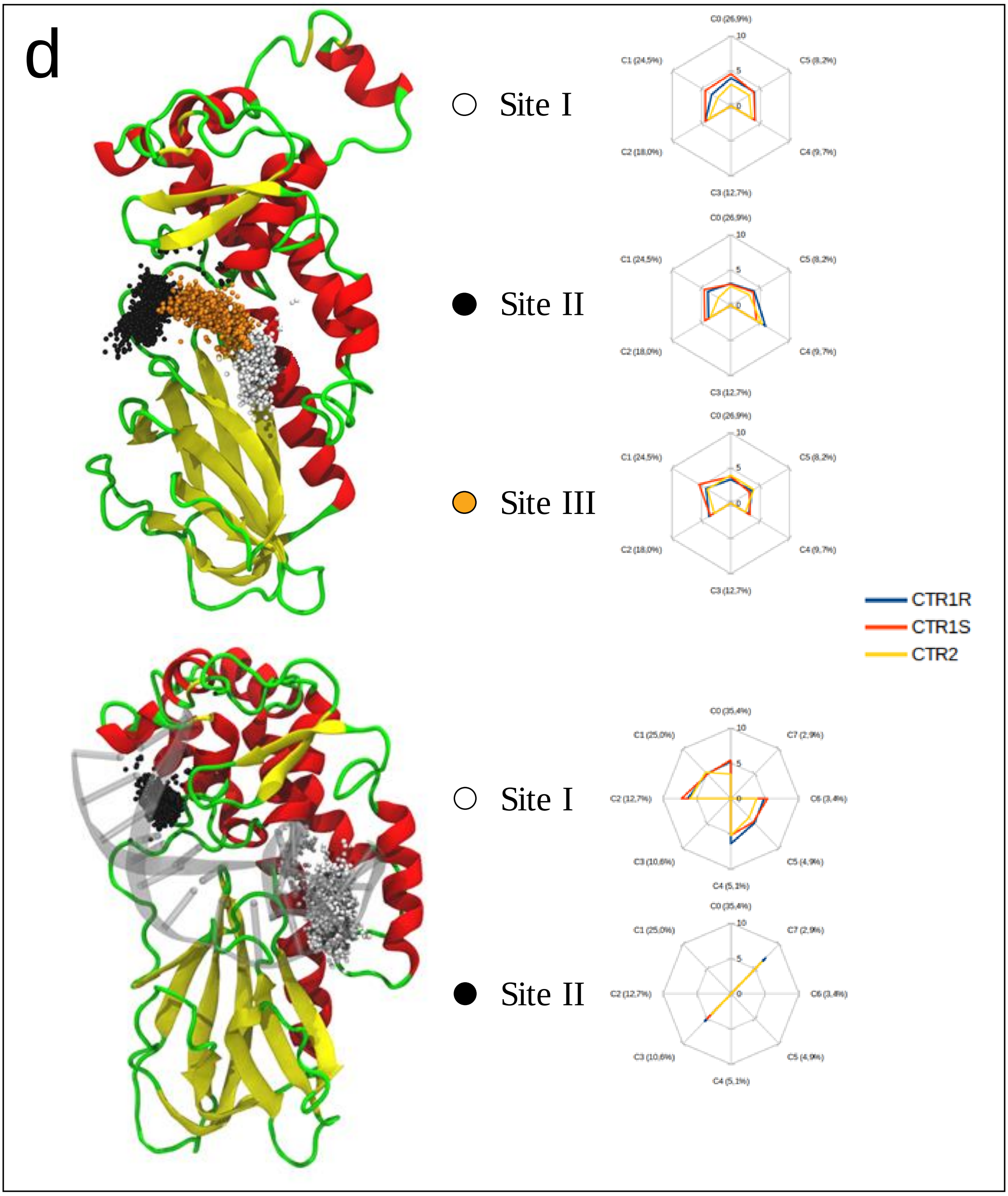

Figure S10d: ***Docking of disulfide forms of TX19 on free and DNA bound hNeil1 visualized on the preferential docking sites I, II and III*** For details, see the Figure S10c legend.

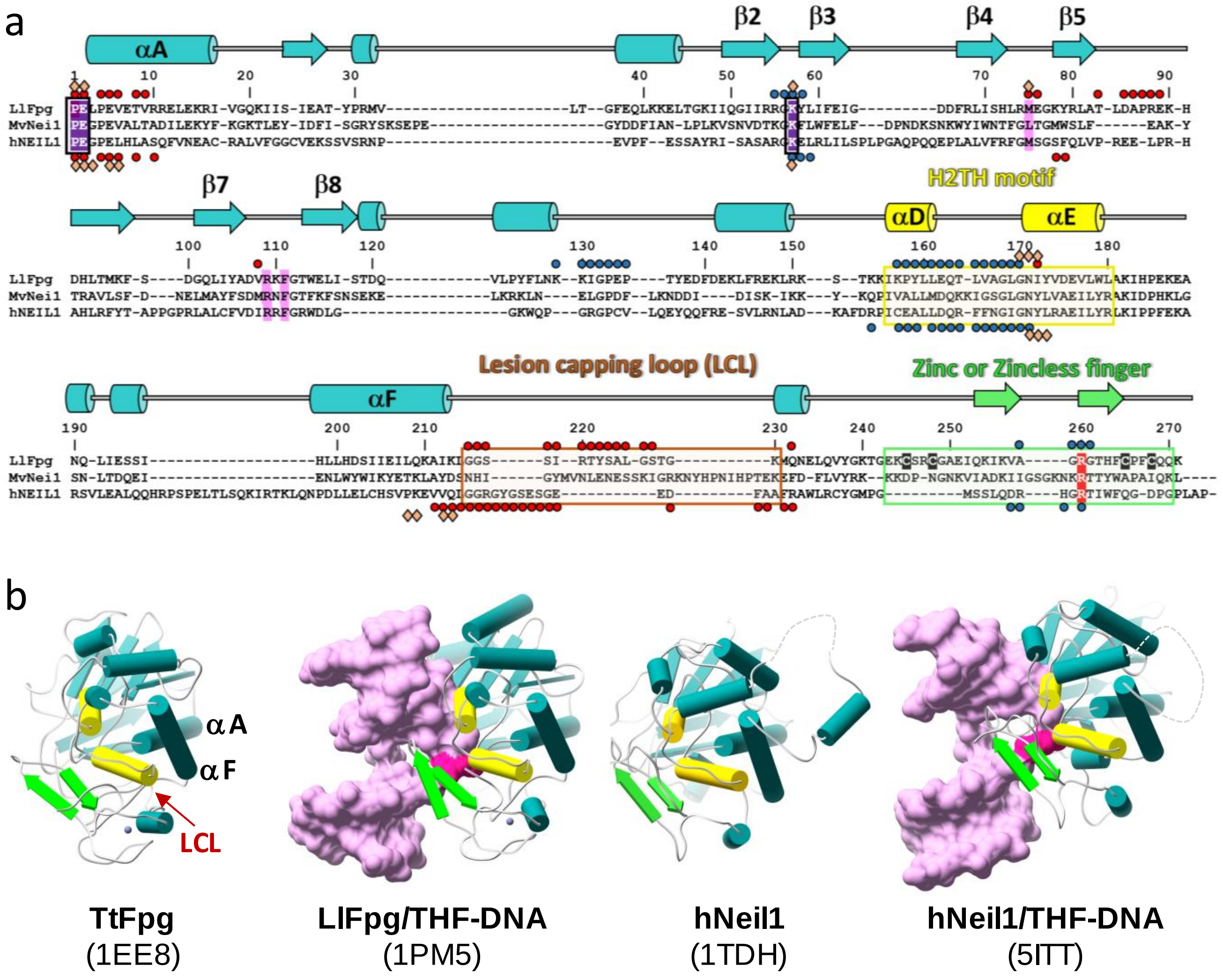

Figure S11. Structures of LlFpg, MvNei1 and hNeil1 ***(a)*** *Primary structure alignment based on 3D-structures* The indicated α-helices (cylinders) and β-strands (arrows) are from LlFpg (observed in 1PM5). P1, E2 and K57 catalytic residues are boxed in *black*. Residues of the intercalation triad are in *black* letters on *light purple* background. The strictly conserved arginine (R) in the *beta* hairpin loop of the zincless and zinc finger is indicated by a *white* letter on a *red* background. The indicated residue numbers correspond to LlFpg. Predicted unstructured, the C-terminal of MvNei1 and hNeil1 proteins has been willingly omitted. The upper (LlFpg) and lower (hNeil1) mall cycles and lozenges highlight residues found by the flexible and blind docking involved in the binding area of the reduced and disulfide forms of 2TX and TX19 (see Table S1 and S2 for more details). *Blue* and *red* cycles are for residues involved in the area of binding sites I and II. *Orange* lozenges indicate residues involved in binding site III. ***(b)*** *Overviews of crystal structures of free or DNA bound Fpg/hNeil1 enzymes used in this study* The Zn^{2+} ion of Fpg zinc finger is indicated by a small *blue* sphere. The H2TH motif and the zinc (or zincless for hNeil1 and MvNei1) finger are highlighted in *yellow* and *green*, respectively. DNA is in *light purple* and THF in *purple*. Ll, h, Tt and Mv are for *Lactococcus lactis*, human, *Thermus thermophilus* and Mimivirus, respectively. The figure is adapted from Boiteux *et al.* (2017) *Free Radical Biology & Medecine* **107**, 179-201.

Figure S12. **2D-LigPlot representations of the best docking poses at site II of reduced and disulfide forms of 2TX and TX19 in DNA bound proteins (a)** and **(b)** are for LlFpg/DNA and hNeil1/DNA complexes, respectively. LlFpg and hNeil1 amino acid residues involved in the interaction with ligands are coloured in *orange* and *green*, respectively. Nucleotides involved in interactions are indicated in *blue* and numbered in accordance with the indicated sequence of DNA duplexes.

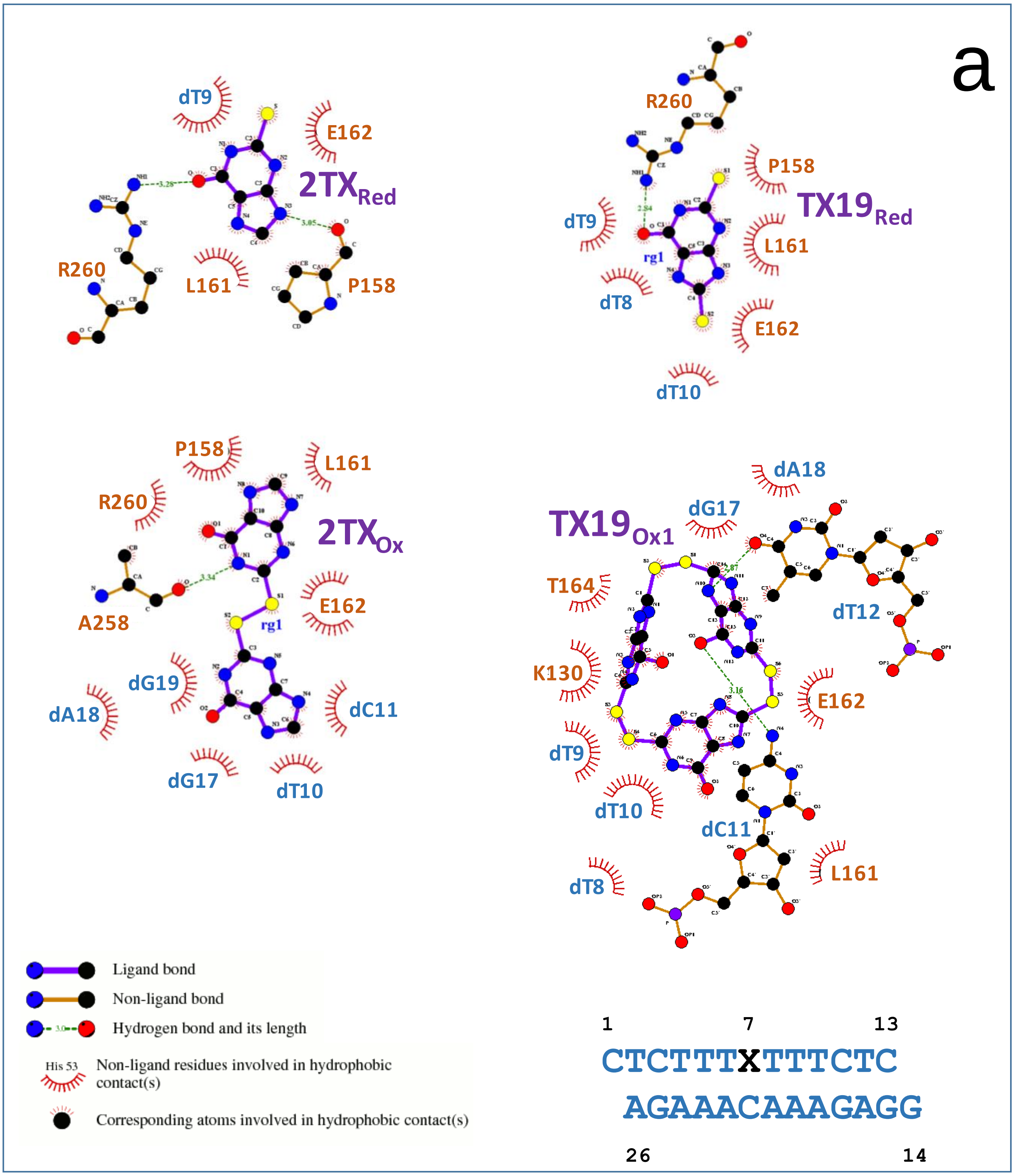

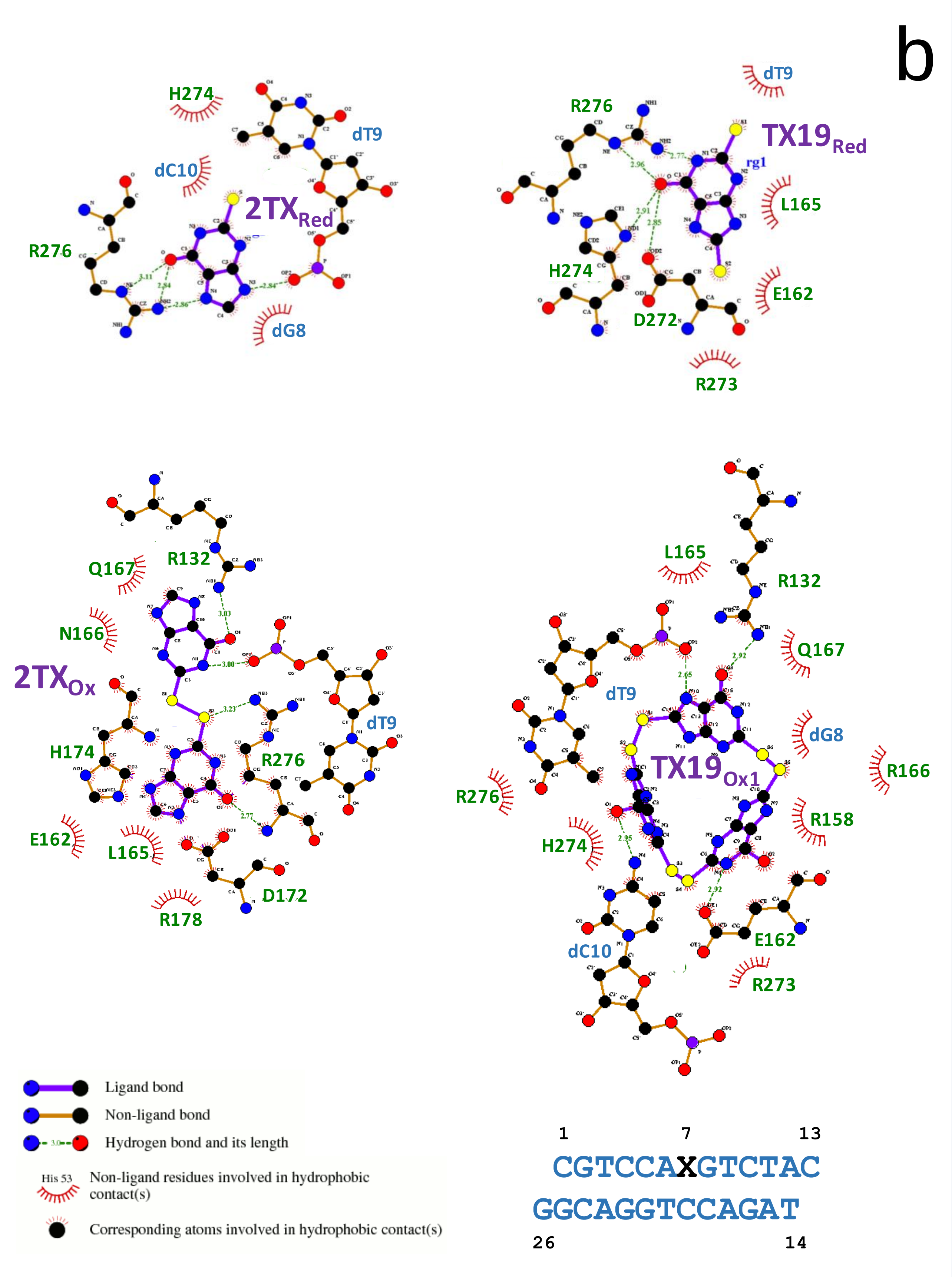
b
2TX_Red
TX19_Red
2TX_Ox
TX19_Ox1
H274
dT9
dC10
R276
dG8
L165
E162
D272
R273
Q167
R132
N166
H174
L165
E162
R178
D172
R166
R158
Ligand bond
Non-ligand bond
Hydrogen bond and its length
His 53
Non-ligand residues involved in hydrophobic contact(s)
Corresponding atoms involved in hydrophobic contact(s)
1 7 13
CGTCCAXGTCTAC
GGCAGGTCCAGAT
26 14

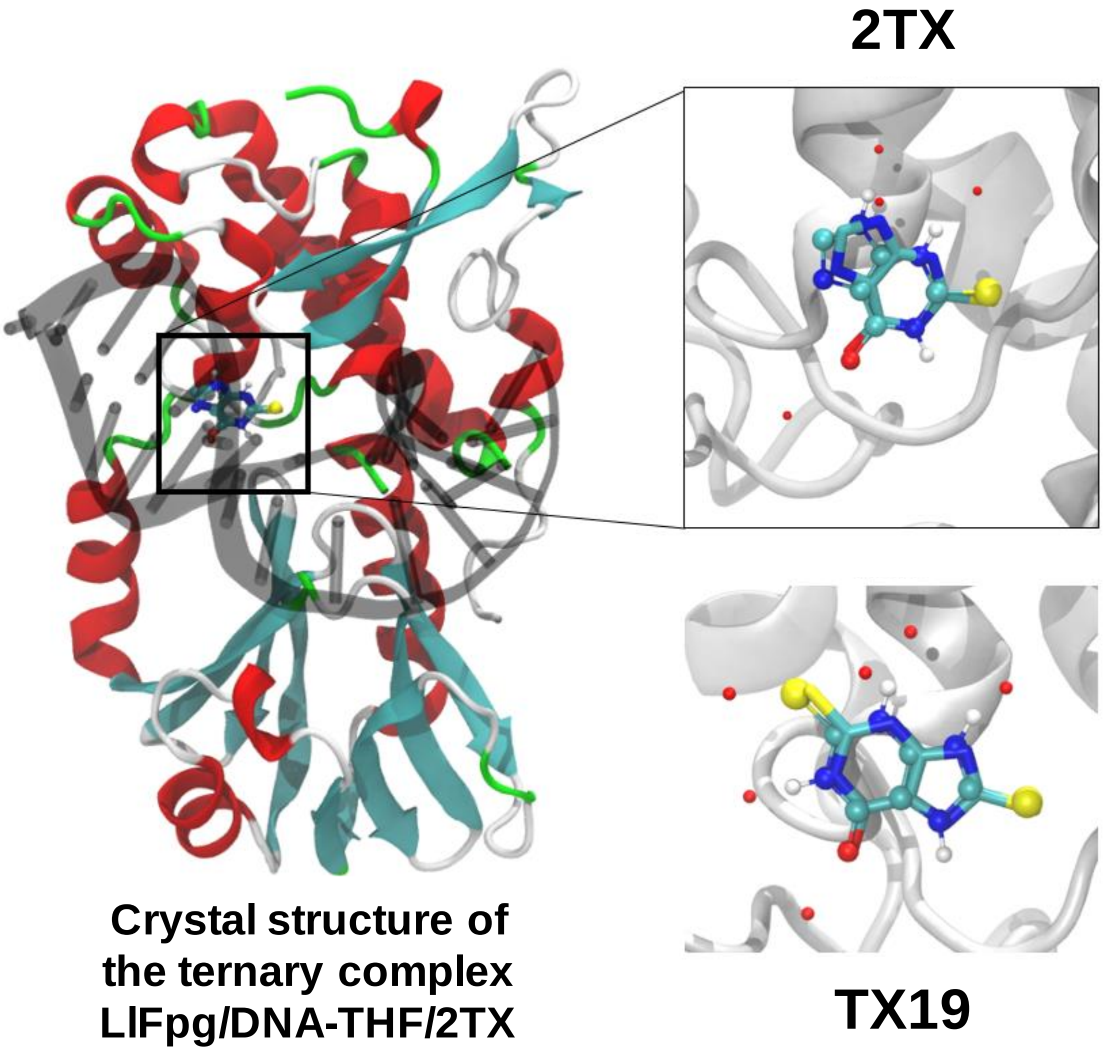

Figure S13. Comparison between crystal structures and docking experiments
Among the poses of 2TX and TX19 having the best docking scores, some of them show a mode of binding at site II that is very similar to that observed in the corresponding crystallographic structures. The selected docking poses of 2TX and TX19 are represented by ball-and-thin sticks and are superimposed on the crystal structures of Fpg/DNA/2TX(TX19) ternary complexes. 2TX and TX19 in the crystal structures are represented by thick sticks. For clarity, the docked Fpg/DNA structure is deliberately omitted. Only docked 2TX and TX19 are represented.
